# Prognostic significance of Lymphocyte-activation gene 3 (LAG3) in patients with solid tumors: a systematic review, meta-analysis and pan-cancer analysis

**DOI:** 10.1186/s12935-023-03157-5

**Published:** 2023-12-02

**Authors:** Rongyang Li, Jianhao Qiu, Zhan Zhang, Chenghao Qu, Zhanpeng Tang, Wenhao Yu, Yu Tian, Hui Tian

**Affiliations:** Department of Thoracic Surgery, Qilu Hospital of Shandong University, Jinan, 250012 Shandong China

**Keywords:** LAG3, Solid tumor, Prognosis, Systematic review, Meta-analysis, Pan-cancer analysis

## Abstract

**Background:**

Lymphocyte-activation gene 3 (LAG3) is a recently discovered immune checkpoint molecule that has been linked to immunosuppression and the advancement of cancer in different types of solid tumors. This study aimed to evaluate the prognostic importance of LAG3 and its role in the immune system within solid tumors.

**Methods:**

Extensive literature searches were conducted using the Pubmed, EMBASE, and Cochrane Library databases to identify relevant studies exploring the effect of LAG3 on survival outcomes. Pooled hazard ratios (HRs) with its 95% confidence intervals (CIs) were calculated to evaluate the prognostic values of LAG3. Afterwards, subgroup analysis and sensitivity analysis were conducted. Pan-cancer analysis investigated the possible relationships between LAG3 expression and genetic alterations, RNA methylation modification-related genes, genomic instability, immune checkpoint genes, and infiltration of immune cells.

**Results:**

A total of 43 studies with 7,118 patients were included in this analysis. Higher expression of LAG3 was associated with worse overall survival (HR = 1.10, 95% CI 1.01–1.19, P = 0.023), but not disease-free survival (HR = 1.41, 95% CI 0.96–2.07, P = 0.078), progression-free survival (HR = 1.12, 95% CI 0.90–1.39, P = 0.317) or recurrence-free survival (HR = 0.98, 95% CI 0.81–1.19, P = 0.871). Subgroup analysis showed that LAG3 might play different prognostic roles in different solid tumors. LAG3 expression was positively associated with immune cell infiltration and immune checkpoint genes in all of the cancers included. LAG3 expression was also found to be associated with microsatellite instability (MSI), copy number variation (CNV), simple nucleoside variation (SNV), tumor mutation burden (TMB), and neoantigen in various types of cancers.

**Conclusions:**

Elevated expression of LAG3 is linked to poorer prognosis among patients diagnosed with solid cancers. LAG3 might play varying prognostic roles in different types of solid tumors. Given its substantial involvement in cancer immunity and tumorigenesis, LAG3 has garnered attention as a promising prognostic biomarker and a potential target for immunotherapy.

**Supplementary Information:**

The online version contains supplementary material available at 10.1186/s12935-023-03157-5.

## Introduction

Accumulating evidence has substantiated the significant involvement of the tumor microenvironment (TME) in the initiation, development, progression, and immune evasion of human tumors [1]. Several immune checkpoint molecules, such as programmed cell death 1 (PD-1) and its ligand (PD-L1), along with cytotoxic T lymphocyte antigen 4 (CTLA-4), have been identified as being connected to the development of an immunosuppressive TME and immune evasion in various types of cancer [2]. During the past few decades, immune checkpoint inhibitors (ICIs) have demonstrated effectiveness in clinical practice for the management of diverse malignancies, resulting in satisfactory therapeutic outcomes [3, 4]. However, the effectiveness of ICIs is limited to a small subset of cancer patients due to either inherent or acquired resistance [4, 5]. Consequently, the identification of novel immune checkpoint molecules, which are correlated with the efficacy of immunotherapy and prognosis, is imperative.

Discovered by Triebel et al. in 1990, Lymphocyte-activation gene 3 (LAG3), also referred to as CD223, is a newly identified immune checkpoint molecule that shares a similar structure to CD4. It is found in activated T cells and natural killer (NK) cells and has been implicated in playing a significant role in immune regulation [6, 7]. LAG3 is expressed on activated CD4^+^ T cells, CD8^+^ T cells and regulatory T cells (Tregs), but also on NK cells, activated B cells and dendritic cells (DCs) [8–10]. It has been widely reported that LAG3 is an inhibitory co-receptor that plays a pivotal role in the dysfunction of antitumor immunity and the formation of an immunosuppressive TME [11]. In recent years, there has been a growing interest in investigating the prognostic significance of LAG3. However, the findings from various studies have yielded conflicting results, leading to a state of controversy in the field.

This study presents a meta-analysis aimed at providing a quantitative summary of the relationship between LAG3 expression and prognosis in individuals diagnosed with solid tumors. Furthermore, a pan-cancer bioinformatic analysis was performed to investigate the correlation between LAG3 expression and the tumor immune microenvironment. This study might provide additional evidence regarding the clinical significance of LAG3 as a prognostic biomarker and as a potential therapeutic target for LAG3-directed immunotherapy.

## Materials and methods

### Meta analysis

#### Protocol and ethics statement

This systematic review and meta-analysis adhered to the guidelines set forth by the Preferred Reporting Items for Systematic Reviews and Meta-Analyses (PRISMA) and the Meta-Analysis of Observational Studies in Epidemiology (MOOSE) [12, 13]. The protocol for this systematic review and meta-analysis has been registered on the INPLASY website (https://inplasy.com/inplasy-2023-8-0073), with the registration number INPLASY202380073. Since the data utilized in this meta-analysis originated from previously published studies, ethical approval and patients' consent were deemed unnecessary for this study.

#### Databases and search strategy

The literature review was conducted by searching three online databases, namely PubMed, EMBASE, and the Cochrane Library, until June 4th, 2023. The search strategy included Medical Subject Headings (MeSH) terms such as "neoplasms" and "LAG3", as well as relevant free terms obtained from PubMed. Two Boolean operators, "AND" and "OR", were used to combine keywords and free terms in all possible combinations. The detailed search strategies for each database can be found in Additional file 7: Table S1. Two authors (Rongyang Li and Jianhao Qiu) independently evaluated and cross-checked the articles. Additionally, the reference lists of excluded publications were manually reviewed to identify any additional viable non-duplicate studies. Any discrepancies between the reviewers were resolved through discussion.

#### Study selection and criteria

The inclusion criteria were as follows: (I) Studies involving patients diagnosed with solid tumors; (II) Studies reporting the expressions of LAG3 in tumor cells and/or tumor-infiltrating lymphocytes (TILs), with patients divided into high and low LAG3 expression groups; (III) Studies providing hazard ratios (HRs) and corresponding 95% confidence intervals (CIs) for LAG3 and survival outcomes; (IV) randomized clinical trials, cohort studies, or case–control studies; (V) Publications in English language.

The exclusion criteria were as follows: (I) Ineligible article types including reviews, meta-analyses, case reports, conference abstracts, letters and comments; (II) Animal studies or basic experimental research; (III) Studies without sufficient data for analyses; (IV) Studies from the same center with patients overlap; (V) Studies published in languages other than English.

#### Data extraction

Two reviewers (Rongyang Li and Jianhao Qiu) conducted an independent assessment of eligible studies and collected the necessary data using predetermined forms. Each study provided the following information: (I) publication details: authors, year of publication, and country; (II) study-related details: study design, analysis method, LAG3 expression level and location, LAG3 cut-off values, methods used to evaluate LAG3 expression, and survival outcomes; (III) demographic information: sample size, cancer type, and treatment; (IV) outcome data: HRs and corresponding 95% CIs for overall survival (OS), disease-free survival (DFS), progression-free survival (PFS), and recurrence-free survival (RFS). The primary objective of this meta-analysis was to assess OS, while DFS, PFS, and RFS served as secondary outcomes. Unpublished data from authors were not sought. In cases where a study included both univariate and multivariate analyses, the results of the multivariate analysis were utilized for further meta-analysis.

#### Quality assessment

The quality of the cohort studies deemed eligible was assessed utilizing the Newcastle–Ottawa Quality Assessment Scale (NOS) [14]. Subsequently, studies with scores of 6 or higher were considered suitable for inclusion in further meta-analysis. Two investigators (Rongyang Li and Jianhao Qiu) independently evaluated the quality of each study, and any discrepancies in the assessment were resolved through discussion.

### Pan-cancer analysis

#### Data preparation

We downloaded the harmonized and standardized pan-cancer dataset: TCGA TARGET GTEx (PANCAN, N = 19,131, G = 60,499) from the UCSC (https://xenabrowser.net/) database [15], then we extracted the expression data of the ENSG00000089692 (LAG3) gene, 44 marker genes for three classes of RNA modifications genes, and 60 marker genes of two classes of immune checkpoint pathways in each sample from solid tissue normal, primary solid tumor, primary tumor, and normal tissue. Furthermore, we performed log_2_(x + 0.001) transformation for each expression value, and we also excluded the cancer types with less than 3 samples in a single cancer type. Finally, the expression data of tumor species mentioned in the meta-analysis were obtained, including colon adenocarcinoma (COAD), bladder urothelial carcinoma (BLCA), colon adenocarcinoma and rectum adenocarcinoma (COADREAD), breast invasive carcinoma (BRCA), cervical squamous cell carcinoma and endocervical adenocarcinoma (CESC), esophageal carcinoma (ESCA), liver hepatocellular carcinoma (LIHC), head and neck squamous cell carcinoma (HNSC), kidney renal clear cell carcinoma (KIRC), lung adenocarcinoma (LUAD), lung squamous cell carcinoma (LUSC), ovarian serous cystadenocarcinoma (OV), rectum adenocarcinoma (READ), pancreatic adenocarcinoma (PAAD), sarcoma (SARC), skin cutaneous melanoma (SKCM), stomach adenocarcinoma (STAD), stomach and esophageal carcinoma (STES), thyroid carcinoma (THCA), and uterine corpus endometrial carcinoma (UCEC).

#### Differential expression analysis

The differences in LAG3 expression levels between normal samples and tumor samples within each type of solid tumor were assessed using the unpaired Wilcoxon-rank sum and signed-rank tests. The results were then visualized using a violin plot.

#### Genetic alteration and RNA methylation modification analysis

The level 4 data set for simple nucleoside variation (SNV) and copy number variation (CNV) of all TCGA samples processed using MuTect2 [16] and GISTIC [17] software were obtained from the GDC database (https://portal.gdc.cancer.gov/). The correlation between LAG3 expression and CNV and SNV was visualized using boxplots. Additionally, the domain information of LAG3 was obtained from the "maftools" package in R software. To illustrate the distribution of protein mutations and their corresponding domains, a lollipop plot was used.

RNA methylation modifications encompass various types such as N1-methyladenosine (m1A), 5-methylcytidine (m5C), and N6-methyladenosine (m6A). These modifications are regulated by genes categorized as writers, readers, and erasers. In this study, we investigated the association between the expression of LAG3 and genes associated with RNA methylation modifications across diverse solid tumor types using spearman correlation analysis.

#### Genomic instability and immune infiltration analysis

Genomic instability was evaluated based on tumor mutation burden (TMB), microsatellite instability (MSI), and neoantigen (NEO) levels. Sangerbox platform (http://www.sangerbox.com/) was used to evaluate the relationship of LAG3 expression with genomic instability in tumors by means of the Spearman correlation coefficient [18].

The infiltration score of tumor-infiltrating immune cells was calculated using the CIBERSORT [19] and TIMER [20] methods, which are available in the "IOBR" package of the R software [21]. In addition, we explored the relationship between LAG3 and 60 immune checkpoint genes using spearman correlation analysis. Heatmap plots were used to visualize the results.

#### Protein − protein interaction network and enrichment analysis

The protein–protein interaction (PPI) network was constructed using the GeneMANIA platform (http://www.genemania.org) [22]. The top 20 genes associated with LAG3 were used to perform Gene Ontology (GO) and Kyoto Encyclopedia of Genes and Genomes (KEGG) enrichment analyses by “clusterProfiler” package in R software. The GO enrichment analyses consisted of terms related to biological process (BP), cellular component (CC), and molecular function (MF).

### Statistical analysis

Pooled HRs and 95% CIs were calculated to assess the prognostic significance of LAG3 in patients with solid tumors. In this study, random-effects models were utilized to calculate pooled effect sizes and reduce potential bias. The heterogeneity level was quantified using the Cochrane Q test and I^2^ statistics, with an I^2^ value greater than 50% indicating substantial heterogeneity [23]. Subgroup analyses were conducted to assess the impact of different cancer types and the location of LAG3 expression. Funnel plots were used to evaluate potential publication bias. Sensitivity analyses were performed to verify the stability of the pooled estimates, wherein the influence of each study on the overall estimates was examined by sequentially excluding individual studies. The statistical significance of the difference between two groups was assessed using the Wilcoxon-rank sum and signed-rank tests, while the Kruskal test was used to examine the difference among multiple groups. The correlation between LAG3 expression and another variable was assessed using Spearman correlation analysis. A 2-sided P value of less than 0.05 was defined as statistical significance. P values below 0.05, 0.01, and 0.001 were denoted as "*", "**", and "***", respectively. All statistical analyzes were conducted using the Stata (version 15.1; Stata Corp, College Station, TX, USA), R version 4.3.1 (R Development Core Team, Vienna, Austria) and Sangerbox platform (http://www.sangerbox.com/) [18].

## Results

### Meta-analysis

#### Literature search

**Fig. 1:**
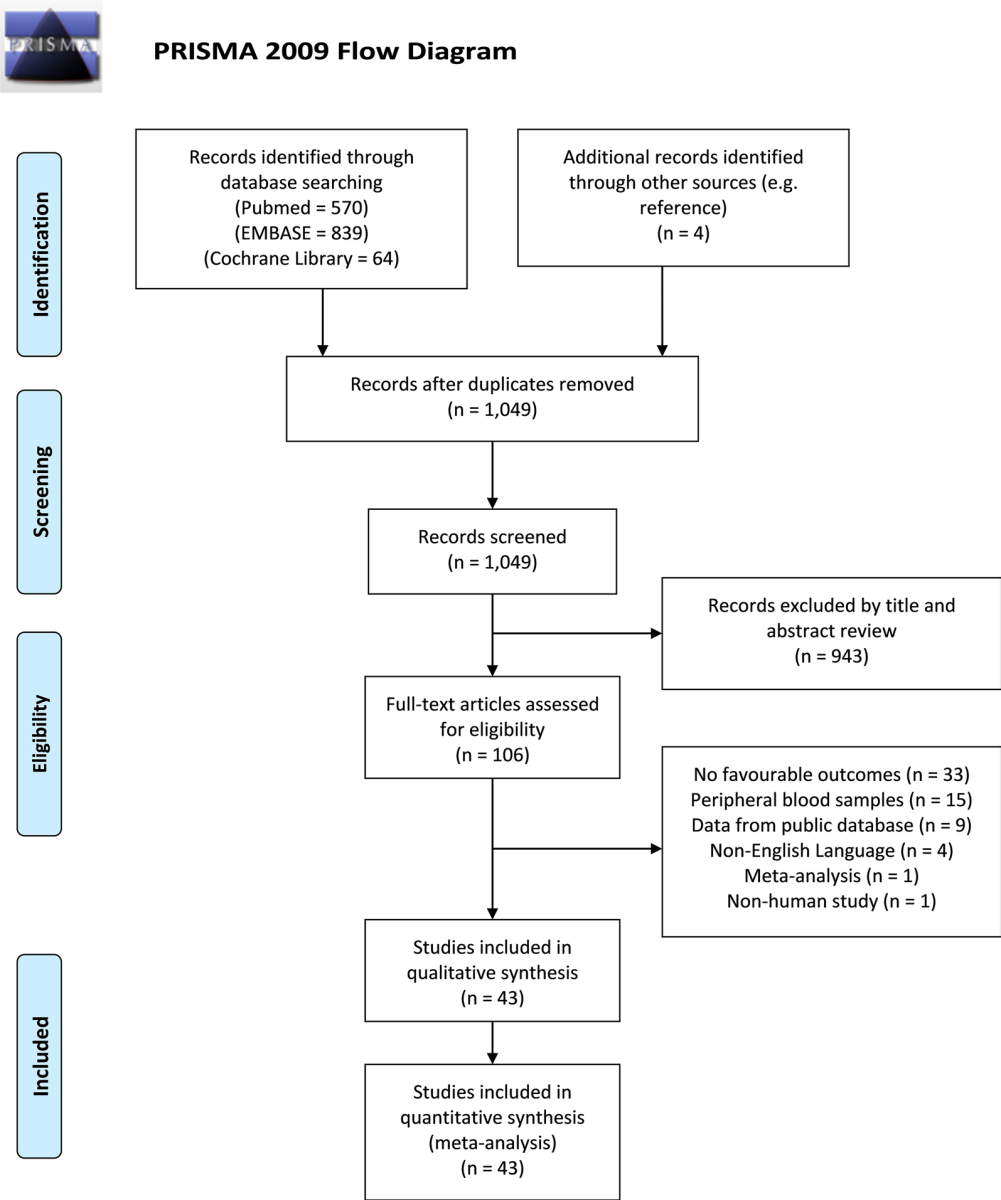
PRISMA flow diagram of literature retrieval. PRISMA, Preferred Reporting Items for Systematic Reviews and Meta-Analyses

A flow diagram outlining the literature search process is presented in Fig. 1. A total of 1,477 potential studies were identified, including 570 from PubMed, 839 from EMBAS, 64 from the Cochrane Library, and 4 relevant studies yielded from the reference list. One thousand and forty-nine studies remained after duplicate publications were removed. After screening of the titles and abstracts of remained studies, nine hundred and forty-three studies were excluded, and the full texts of 106 studies were reviewed. Finally, forty-three studies with 7,118 patients were included in this meta-analysis [24–66].

#### Characteristics of the included studies

The baseline characteristics and methodological assessment of included studies are presented in Table 1. All 43 studies were retrospective cohort studies conducted in 12 countries around the world. The publication dates of the included studies ranged from 2015 to 2023, and the sample sizes of the included studies ranged from 25 to 564. Eight studies reported the survival outcome of BRCA [24, 26, 27, 51, 54–56, 60], seven studies reported the survival outcome of ESCA [25, 28, 30, 32, 59, 61, 64], five studies reported the survival outcome of HNSC [29, 43, 46, 50, 57], and three studies reported the survival outcome of OV [31, 37, 62]. Two studies each explored the survival outcome of patients with BLCA [36, 63], COAD [39, 49], LIHC [34, 42], THCA [44, 53], STAD [45, 47], and SKCM [38, 40]. Only one study each reported the survival outcome of non-small cell lung cancer (NSCLC) [35], CESC [66], KIRC [33], SARC [41], READ [48], COADREAD [58], PAAD [52], and UCEC [65]. Thirty-two of the included studies reported the correlation between LAG3 expression and OS [24–35, 37, 38, 40, 42, 44–47, 50, 51, 54–59, 62–64, 66], fourteen reported the correlation LAG3 expression and DFS [24–26, 33, 34, 39, 42, 43, 45, 46, 48, 49, 52, 60], ten reported the correlation between LAG3 expression and PFS [37, 38, 41, 47, 50, 61–64, 66], and eight reported the correlation between LAG3 expression and RFS [27, 31, 35, 36, 53, 55, 59, 65]. The NOS scores of included studies ranged from 7 to 9, indicating the overall quality was high. The detailed quality assessment is presented in Additional file 8: Table S2.

**Table 1:** Baseline characteristics of included studies regarding LAG3

Author (year)	Country	Sample size	Study type	Cancer type	Treatment	LAG3 + expression	Expression location	Cut-off value of LAG3	Method to evaluate LAG3 expression	Outcome	Method to estimate HR
Asano (2022)	Japan	177	RCS	BRCA	NAC + Surgery	47 (26.6)	TILs	NR	IHC + FISH	OS	Univariate
										DFS	Multivariate
Babar (2019)	USA	49	RCS	ESCA	NAC/NRT + Surgery	NR	TCs	> 2.9759	IHC	OS	Univariate
								> 3.7521		DFS	Multivariate
Bagbudar (2022)	Turkey	238	RCS	BRCA	Surgery	179 (75.2)	TILs	> 1%	IHC	OS	Multivariate
										DFS	Univariate
Bottai (2016)	Italy	363	RCS	BRCA	AC	NR	TILs	> 5%	IHC	OS	Univariate
										RFS	Univariate
Chen (2021)	China	161	RCS	ESCA	Surgery	92 (57.1)	TCs	≥ 10%	IHC	OS	Univariate
Deng (2016)	China	122	RCS	HNSC	NAC/NRT + Surgery	NR	TILs	NR	IHC + IF	OS	Univariate
Duan (2018)	China	95	RCS	ESCA	Surgery	16 (16.8)	TILs	≥ 1%	IHC + IF	OS	Univariate
Fucikova (2019)	Czech	80	RCS	OV	Surgery	40 (50.0)	TCs	Median level	IHC	OS	Univariate
										RFS	Univariate
Gebauer (2020)	Germany	421	RCS	ESCA	Surgery or NAC/NRT + Surgery	48 (11.4)	TILs	≥ 1%	IHC + IF	OS	Multivariate
Giraldo (2015)	France	135	RCS	KIRC	Surgery	NR	Tumor Center TILs	> 5%	IHC + IF	OS	Univariate
										DFS	Univariate
							Invasive Margin TILs			OS	Univariate
										DFS	Univariate
Guo (2020)	China	143	RCS	LIHC	Surgery	60 (42.0)	TCs	≥ 4.9/mm2	IF	OS	Multivariate
										DFS	Multivariate
He (2017)	China	139	RCS	NSCLC	Surgery	36 (25.9)	TILs	> 20%	IHC	OS	Multivariate
										RFS	Multivariate
Jin (2023)	China	175	RCS	BLCA	Surgery	18 (10.8)	TCs	CPS ≥ 1	IHC	RFS	Multivariate
Kim (2018)	Korea	108	RCS	OV	NAC + Surgery + AC	46 (41.8)	TILs	Median level	IHC	OS	Univariate
										PFS	Univariate
Kim (2020)	Korea	102	RCS	SKCM	Skin biopsy	44 (43.1)	TAMs	> 20%	IHC	OS	Multivariate
										PFS	Multivariate
Lee (2018)	Korea	89	RCS	COAD	Surgery	44 (49.4)	TILs	> 5%	IHC	DFS	Multivariate
Lee (2019)	Korea	124	RCS	SKCM	Skin biopsy	55 (44.4)	TILs	> 20%	IHC + IF	OS	Multivariate
Ligon (2021)	USA	25	RCS	SARC	Surgery or Surgery + AC	NR	TCs	NR	IHC + FC	PFS	Univariate
Luo, C (2021)	China	31	RCS	LIHC	Surgery	NR	TCs	> 5%	IHC	OS	Univariate
										DFS	Univariate
Luo, F (2021)	China	182	RCS	HNSC	Surgery + AC/RT/AC + RT	147 (80.8)	TILs	> 14/mm2	IHC	DFS	Multivariate
Luo (2022)	China	113	RCS	THCA	Surgery + I_131_/AC/RT/AC + RT	NR	TILs	CPS ≥ 1	IHC	OS	Univariate
Lv (2021)	China	564	RCS	STAD	Surgery	228 (40.4)	TILs	Median level	IHC	OS	Univariate
										DFS	Univariate
Minichsdorfer (2019)	Austria	28	RCS	HNSC	Biopsy/Surgery + AC + RT	15 (53.6)	TILs	> 5%	IHC	OS	Univariate
										DFS	Univariate
Park (2021)	Korea	385	RCS	STAD	Surgery + AC	175 (45.5)	TCs	≥ 5%	IHC	OS	Multivariate
						25 (6.5)	TILs			OS	Univariate
						29 (7.5)				PFS	Univariate
Peng (2021)	China	76	RCS	READ	NRT + Surgery + AC	28 (36.8)	TCs	> 12.5%	IHC	DFS	Univariate
						26 (34.2)	TILs	> 27.5%		DFS	Multivariate
Rhyner Agocs (2021)	Switzerland	142	RCS	COAD	Surgery	98 (69.0)	TILs	> 1/mm2	IHC	DFS	Univariate
Rühle (2022)	Germany	63	RCS	HNSC	AC/RT	NR	Intraepithelial TILs	CPS ≥ 1	IHC	OS	Univariate
										PFS	Univariate
							Stromal TILs			OS	Univariate
										PFS	Univariate
Sarradin (2021)	France	66	RCS	BRCA	NAC + Surgery	55 (83.3)	TCs	≥ 1%	IHC	OS	Univariate
Seifert (2021)	Germany	58	RCS	PAAD	Surgery or NAC + Surgery	29 (50.0)	TILs	Median level	IHC + IF	DFS	Multivariate
Shi (2021)	China	200	RCS	THCA	Surgery	6 (3.0)	TILs	CPS ≥ 1	IHC	RFS	Univariate
Stovgaard (2021)	Denmark	225	RCS	BRCA	Surgery + AC	37 (16.4)	intratumoral TILs	CPS ≥ 1	IHC	OS	Univariate
						82 (36.4)	stromal TILs			OS	Univariate
Stovgaard (2022)	Denmark	488	RCS	BRCA	Surgery + AC	227 (46.5)	intratumoral TILs	Median level	IHC	OS	Multivariate
										RFS	Multivariate
						154 (31.6)	stromal TILs			OS	Multivariate
										RFS	Multivariate
Tahtacı (2023)	Turkey	49	RCS	BRCA	NAC + Surgery + AC/RT	28 (57.1)	stromal TILs	> 1%	IHC	OS	Univariate
Wang, H (2019)	China	36	RCS	HNSC	TT	NR	TILs	IHC scoring > 4	IHC	OS	Multivariate
Wang, W (2019)	China	183	RCS	ESCA	Surgery	69 (37.7)	TILs	Scoring system > 3	IHC	OS	Multivariate
										RFS	Multivariate
Wang (2018)	China	114	RCS	BRCA	NAC + Surgery + AC	38 (33.3)	TILs	> 5%	IHC	DFS	Univariate
Wang (2021)	China	96	RCS	COADREAD	Surgery + AC	8 (8.3)	TCs	> 1%	IHC	OS	Univariate
Yao (2023)	China	78	RCS	ESCA	NAC + Surgery	26 (33.3)	TILs	NR	RT-PCR + WB	PFS	Multivariate
Zaitsu (2023)	Japan	171	RCS	OV	Surgery	48 (28.1)	TILs	> 20%	IHC	OS	Univariate
										PFS	Multivariate
Zeng (2020)	China	141	RCS	BLCA	Surgery + AC	71 (50.4)	intraepithelial TILs	≥ 10 cells/HPF	IHC + FC	OS	Multivariate
										PFS	Multivariate
						68 (48.2)	stromal TILs	≥ 1 cells/HPF		OS	Multivariate
										PFS	Multivariate
Zhang (2018)	China	287	RCS	ESCA	Surgery	172 (59.9)	TILs	Median level	IHC	OS	Multivariate
										PFS	Multivariate
Zhang (2022)	China	421	RCS	UCEC	Surgery	133 (31.6)	TCs	> 1%	IHC	RFS	Multivariate
						101 (24.0)	TILs			RFS	Univariate
Zou (2023)	China	175	RCS	CESC	NAC + NRT + Surgery or Surgery + CRT/RT	50 (28.6)	TCs	CPS ≥ 1	IHC	OS	Univariate
										PFS	Univariate
						18 (10.3)		CPS ≥ 10		OS	Univariate
										PFS	Univariate

RCS, retrospective cohort study; NR, not reported; LAG3, lymphocyte- activation gene 3; HR, hazard ratio; BLCA, bladder urothelial carcinoma; BRCA, breast invasive carcinoma; CESC, cervical squamous cell carcinoma and endocervical adenocarcinoma; COAD, colon adenocarcinoma; COADREAD, colon adenocarcinoma or/and rectum adenocarcinoma; ESCA, esophageal carcinoma; HNSC, head and neck squamous cell carcinoma; KIRC, kidney renal clear cell carcinoma; LIHC, liver hepatocellular carcinoma; NSCLC, non-small cell lung carcinoma; OV, ovarian serous cystadenocarcinoma; PAAD, pancreatic adenocarcinoma; READ, rectum adenocarcinoma; SARC, sarcoma; SKCM, skin cutaneous melanoma; STAD, stomach adenocarcinoma; THCA, thyroid carcinoma; UCEC, uterine corpus endometrial carcinoma; NAC, neoadjuvant chemotherapy; NRT, neoadjuvant radiation therapy; AC, adjuvant chemotherapy; RT, radiation therapy; TT, targeted therapy; CRT: chemoradiotherapy; TCs, tumor cells; TILs, tumor-infiltrating lymphocytes; TAMs, tumor-associated macrophages; CPS, combined positive score; HPF, high-power field; IHC, immunohistochemical; IF, immunofluorescence; FC, flow cytometry; FISH, fluorescence in situ hybridization; RT-PCR, reverse transcription polymerase chain reaction; WB, Western Blotting; OS, overall survival; DFS, disease-free survival; PFS, progression-free survival; RFS, recurrence-free survival

#### Overall survival

**Fig. 2:**
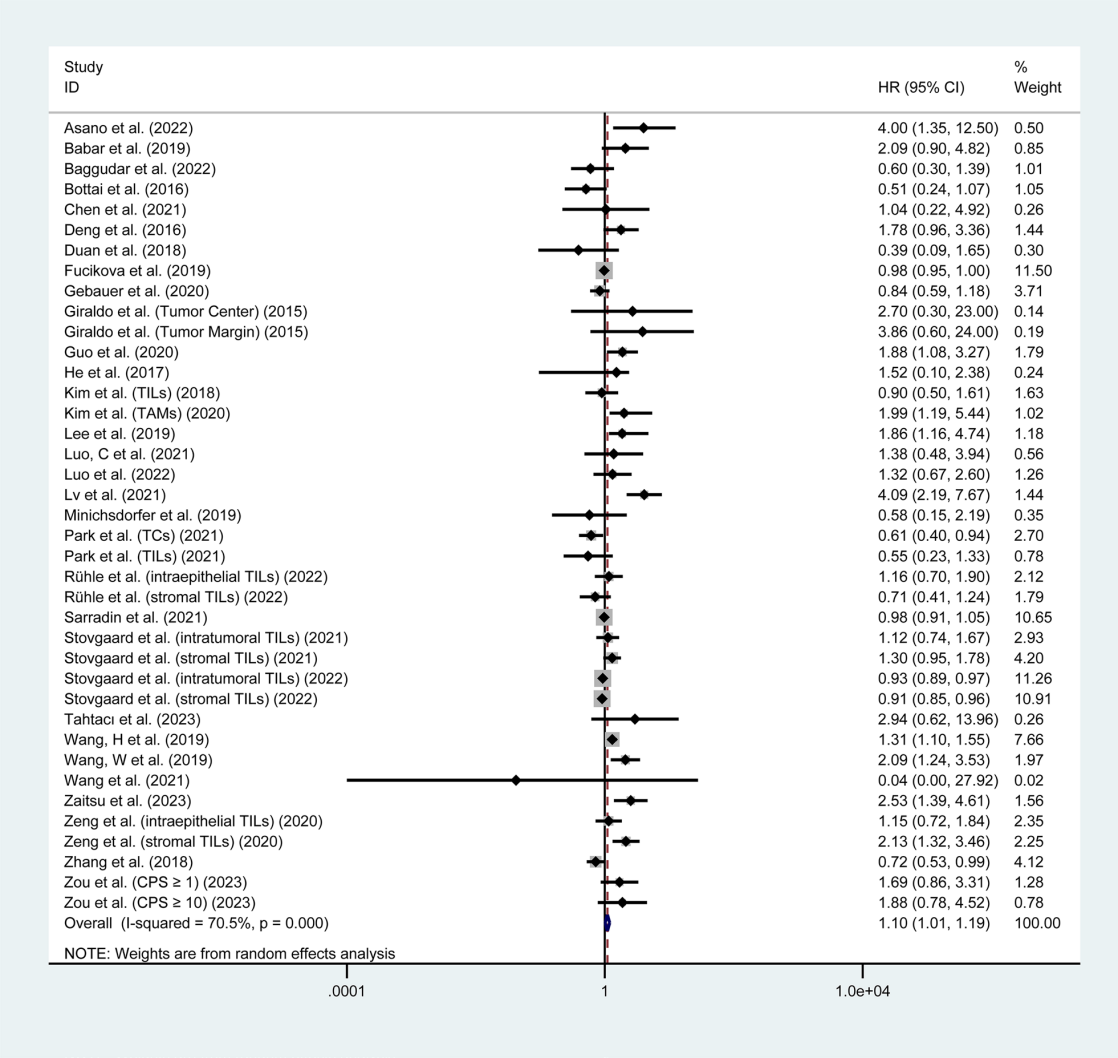
Forest plot of the correlation between LAG3 and overall survival (OS) in patients with solid tumors. HR, hazard ratio; CI, confidence interval

Thirty-two studies involving 5,558 patients investigated the correlation between LAG3 expression and OS [24–35, 37, 38, 40, 42, 44–47, 50, 51, 54–59, 62–64, 66]. The pooled analysis indicated that increased expression of LAG3 was associated with worse OS in patients with solid tumors (HR = 1.10, 95% CI 1.01–1.19, P = 0.023), with significant heterogeneity (I^2^ = 70.5%, P < 0.001), as shown in Fig. 2. To further investigate the prognostic significance of LAG3, we performed subgroup analyses according to cancer types and the location of LAG3 expression. The results of subgroup analyses of OS are presented in Table 2 and Additional file 1: Fig. S1. Subgroup analysis based on tumor type showed that higher expression of LAG3 was correlated with worse OS in patients with CESC (HR = 1.76, 95% CI 1.03–3.00, P = 0.038), LIHC (HR = 1.76, 95% CI 1.08–2.87, P = 0.024), and SKCM (HR = 1.92, 95% CI 1.15–3.22, P = 0.013). There was no significant prognostic value of LAG3 expression found in the OS for BLCA, BRCA, COADREAD, ESCA, HNSC, OV, KIRC, NSCLC, THCA, and STAD (P > 0.05). Additionally, we found that higher LAG3 expression in tumor-infiltrating lymphocytes (TILs) was associated with shorter OS (HR = 1.16, 95% CI 1.02–1.31, P = 0.021). However, there was no significant correlation between LAG3 expression in tumor cells (TCs) or tumor-associated macrophages (TAMs) and OS (P > 0.05).

**Table 2:** Subgroup analysis of the correlation between LAG3 expression and survival outcomes in patients with solid tumors

Categories	OS					DFS					PFS					RFS				
	Random effects model		Heterogeneity		P	Random effects model		Heterogeneity		P	Random effects model		Heterogeneity		P	Random effects model		Heterogeneity		P
	HR	95% CI	I^2^	P		HR	95% CI	I^2^	P		HR	95% CI	I^2^	P		HR	95% CI	I^2^	P	
Total	1.1	1.01–1.19	70.5%	< 0.001	0.023	1.41	0.96–2.07	77.5%	< 0.001	0.078	1.12	0.90–1.39	64.7%	0.001	0.317	0.98	0.81–1.19	70.3%	< 0.001	0.871
cancer type																				
BLCA	1.56	0.85–2.86	69.0%	0.072	0.148						1.58	1.12–2.23	0.0%	0.339	0.01	1.97	0.80–4.83	-	-	0.139
BRCA	0.96	0.89–1.04	59.8%	0.011	0.328	1.32	0.43–4.08	89.1%	< 0.001	0.632						0.87	0.78–0.97	54.3%	0.012	0.01
CESC	1.76	1.03–3.00	0.0%	0.853	0.038						1.16	0.69–1.97	0.0%	0.907	0.569					
COAD						0.31	0.15–0.64	0.0%	0.707	0.001										
COADREAD	0.04	0.00–22.19	–	–	0.322															
ESCA	1.07	0.68–1.69	70.4%	0.005	0.767	2.86	1.03–7.94	–	–	0.044	1.47	0.30–7.13	87.2%	0.005	0.632	1.72	1.06–2.79	–	–	0.028
HNSC	1.16	0.87–1.54	41.8%	0.143	0.316	1.73	0.83–3.61	57.0%	0.127	0.141	0.82	0.57–1.18	0.0%	0.355	0.276					
KIRC	3.32	0.82–13.55	0.0%	0.806	0.094	8.92	2.40–33.18	15.7%	0.276	0.001										
LIHC	1.76	1.08–2.87	0.0%	0.608	0.024	1.62	1.08–2.41	0.0%	0.856	0.019										
NSCLC	1.52	0.30–7.60	-	-	0.612											1.55	0.99–2.42	–	–	0.056
OV	1.25	0.73–2.14	79.3%	0.008	0.419						1.39	0.87–2.23	35.2%	0.214	0.168	0.5	0.29–0.85	–	–	0.01
PAAD						1.94	1.20–3.14	–	–	0.007										
READ						0.73	0.42–1.28	0.0%	0.987	0.269										
SARC											1.1	1.02–1.19	–	–	0.016					
SKCM	1.92	1.15–3.22	0.0%	0.898	0.013						1.87	0.88–3.99	–	–	0.106					
STAD	1.12	0.31–4.12	92.3%	< 0.001	0.861	2.65	1.53–4.58	–	–	< 0.001	0.27	0.11–0.68	–	–	0.005					
THCA	1.32	0.67–2.60			0.422											1.01	0.14–7.31	–	–	0.992
UCEC																2.11	0.41–10.77	69.3%	0.071	0.369
Expression location																				
TCs	1.02	0.91–1.14	52.6%	0.025	0.772	1.43	0.86–2.37	47.5%	0.126	0.171	1.1	1.02–1.19	0.0%	0.972	0.014	1.52	0.40–5.82	84.5%	0.002	0.539
TILs	1.16	1.02–1.31	74.2%	< 0.001	0.021	1.4	0.85–2.32	81.9%	< 0.001	0.184	1.08	0.76–1.54	74.4%	< 0.001	0.664	0.97	0.82–1.15	65.4%	0.008	0.72
TAMs	1.99	0.93–4.25	–	–	0.076						1.87	0.88–3.99	–	–	0.106					

LAG3, lymphocyte- activation gene 3; CI, confidence interval; DFS, disease-free survival; OS, overall survival; PFS, progression-free survival; RFS, recurrence-free survival; BLCA, bladder urothelial carcinoma; BRCA, breast invasive carcinoma; CESC, cervical squamous cell carcinoma and endocervical adenocarcinoma; COAD, colon adenocarcinoma; COADREAD, colon adenocarcinoma or/and rectum adenocarcinoma; ESCA, esophageal carcinoma; HNSC, head and neck squamous cell carcinoma; KIRC, kidney renal clear cell carcinoma; LIHC, liver hepatocellular carcinoma; NSCLC, non-small cell lung carcinoma; OV, ovarian serous cystadenocarcinoma; PAAD, pancreatic adenocarcinoma; READ, rectum adenocarcinoma; SARC, sarcoma; SKCM, skin cutaneous melanoma; STAD, stomach adenocarcinoma; THCA, thyroid carcinoma; UCEC, uterine corpus endometrial carcinoma; TCs, tumor cells; TILs, tumor-infiltrating lymphocytes; TAMs, tumor-associated macrophages

#### Disease-free survival

**Fig. 3:**
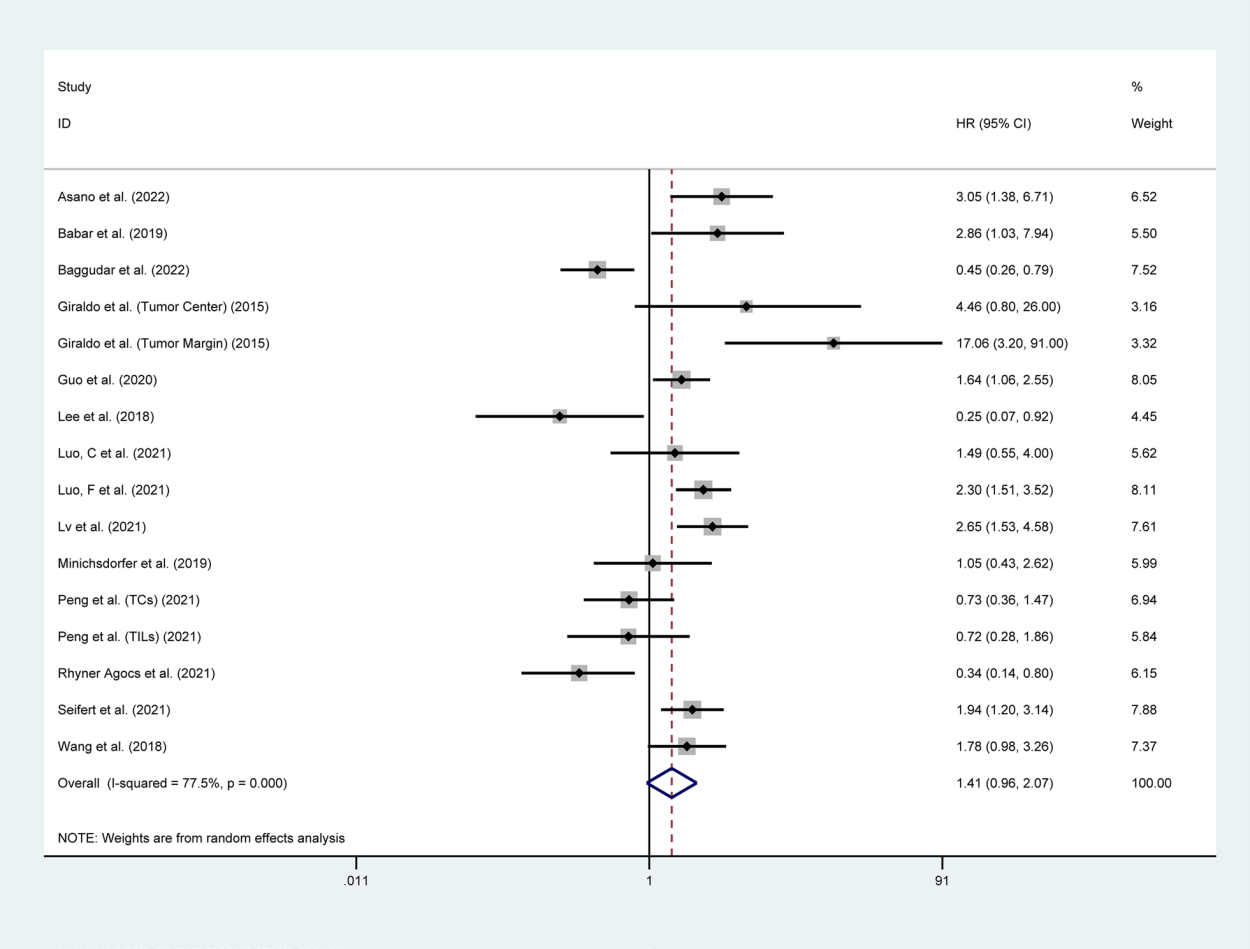
Forest plot of the correlation between LAG3 and disease-free survival (DFS) in patients with solid tumors. HR, hazard ratio; CI, confidence interval

Fourteen studies involving 2,026 patients investigated the correlation between the expression of LAG3 and DFS [24–26, 33, 34, 39, 42, 43, 45, 46, 48, 49, 52, 60]. The pooled analysis showed that there was no significant correlation between LAG3 expression and DFS (HR = 1.41, 95% CI 0.96–2.07, P = 0.078), as shown in Fig. 3. Subgroup analysis based on tumor type indicated that higher expression of LAG3 was correlated with worse DFS in patients with ESCA (HR = 2.86, 95% CI 1.03–7.94, P = 0.044), KIRC (HR = 8.92, 95% CI 2.40–33.18, P = 0.001), LIHC (HR = 1.62, 95% CI 1.08–2.41, P = 0.019), PAAD (HR = 1.94, 95% CI 1.20–3.14, P = 0.007), and STAD (HR = 2.65, 95% CI 1.53–4.58, P < 0.001). However, increased expression of LAG3 was associated with better DFS in patients with COAD (HR = 0.31, 95% CI 0.15–0.64, P = 0.001). There was no significant prognostic value of LAG3 expression found in the DFS for BRCA, HNSC, and READ (P > 0.05). Additionally, there was no significant correlation between LAG3 expression in TCs or TILs and DFS (P > 0.05) (Table 2 and Additional file 2: Figure S2).

#### Progression-free survival

**Fig. 4:**
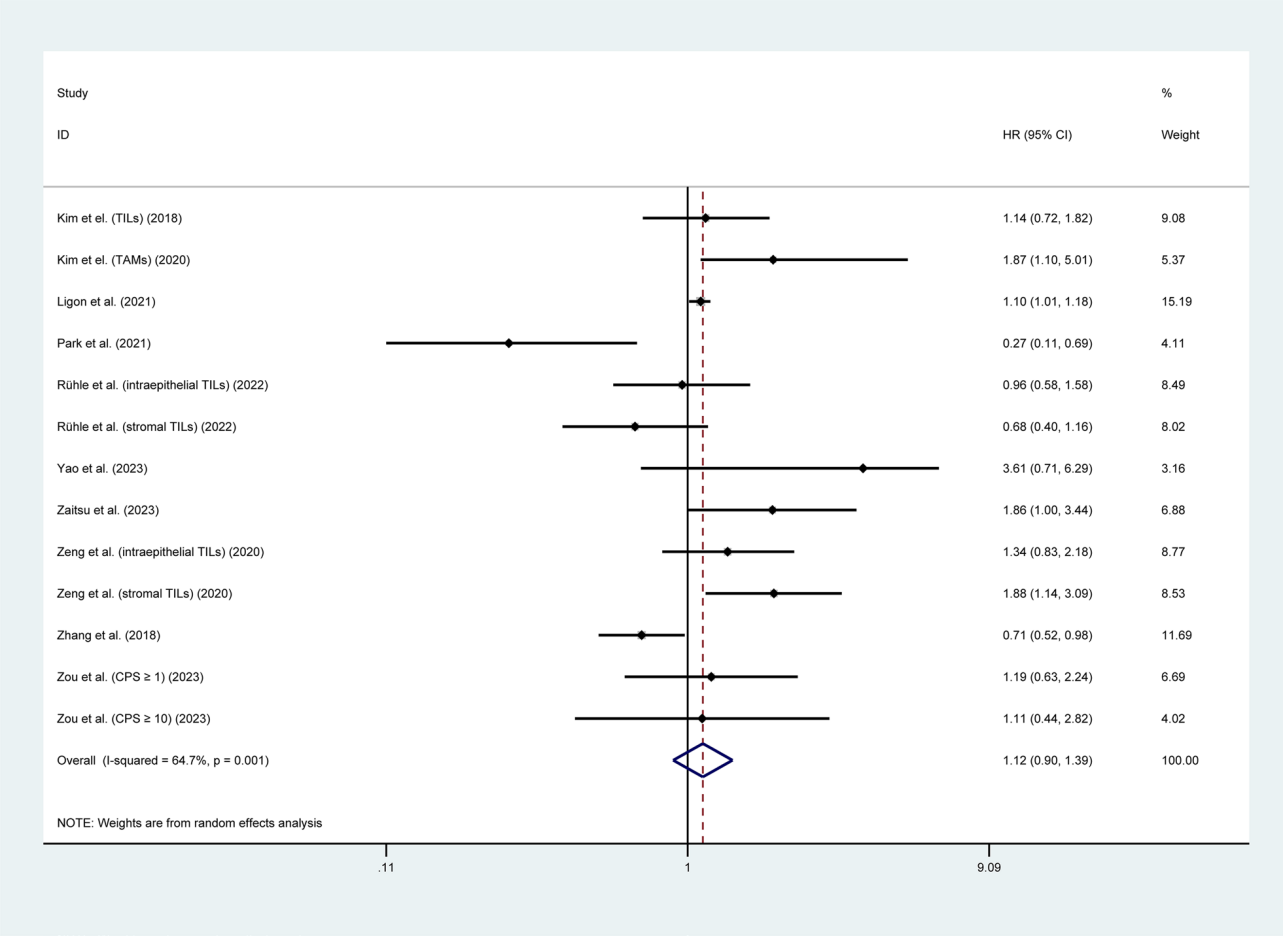
Forest plot of the correlation between LAG3 and progression-free survival (PFS) in patients with solid tumors. HR, hazard ratio; CI, confidence interval

Ten studies involving 1,535 patients investigated the correlation between the expression of LAG3 and PFS [37, 38, 41, 47, 50, 61–64, 66]. The pooled analysis showed that there was no significant correlation between LAG3 expression and PFS (HR = 1.12, 95% CI 0.90–1.39, P = 0.317), as shown in Fig. 4. Subgroup analysis based on tumor type indicated that higher expression of LAG3 was correlated with worse PFS in patients with BLCA (HR = 1.58, 95% CI 1.12–2.23, P = 0.010) and SARC (HR = 1.10, 95% CI 1.02–1.19, P = 0.016). However, increased expression of LAG3 was associated with better PFS in patients with STAD (HR = 0.27, 95% CI 0.11–0.68, P = 0.005). There was no significant prognostic value of LAG3 expression was found in the PFS for CESC, ESCA, HNSC, OV and SKCM (P > 0.05). Additionally, we found that higher LAG3 expression in TCs was associated with shorter PFS (HR = 1.10, 95% CI 1.02–1.19, P = 0.014), while there was no significant correlation between LAG3 expression in TILs or TAMs and PFS (P > 0.05) (Table 2 and Additional file 3: Figure S3).

#### Recurrence-free survival

**Fig. 5:**
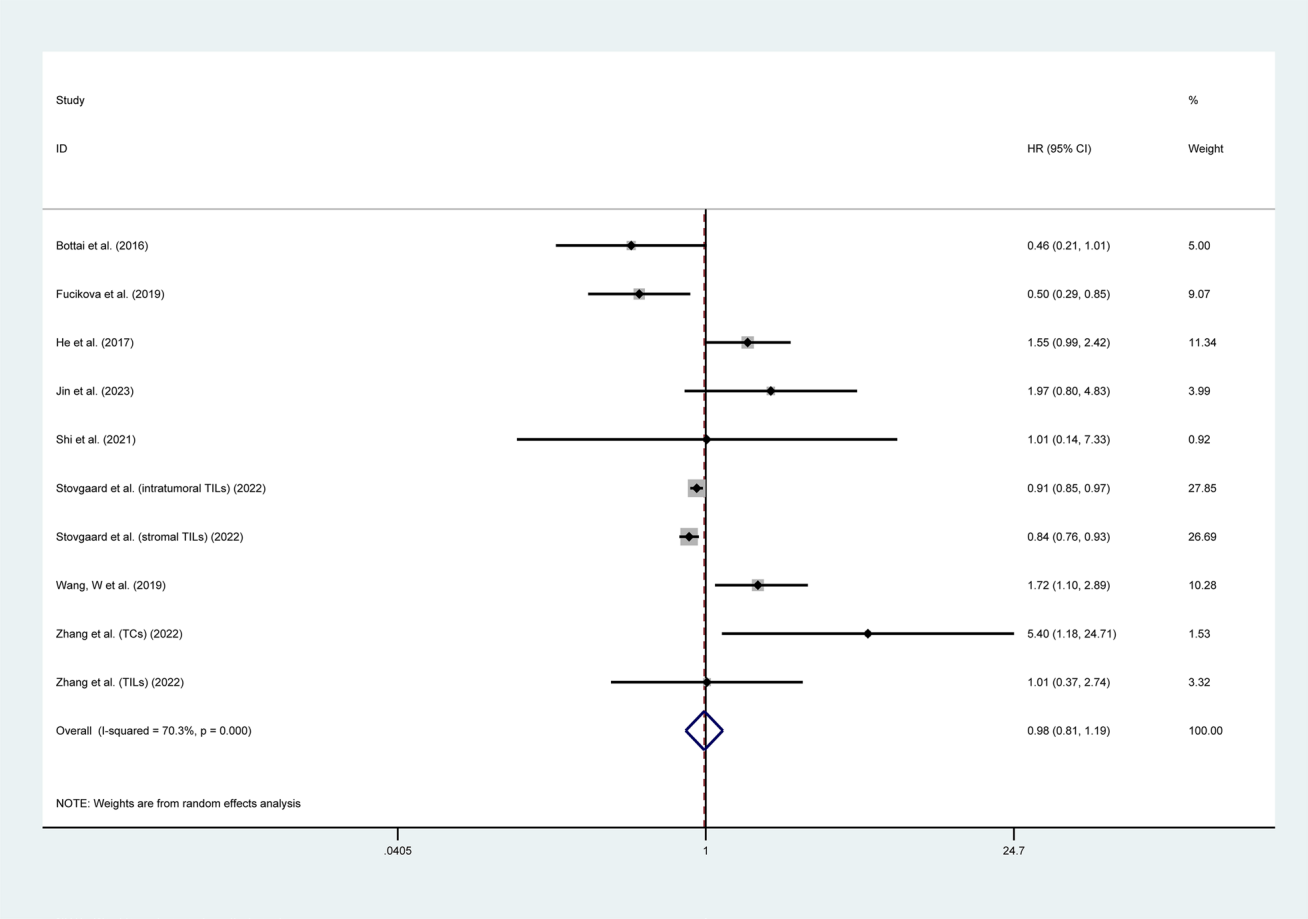
Forest plot of the correlation between LAG3 and recurrence-free survival (RFS) in patients with solid tumors. HR, hazard ratio; CI, confidence interval

Eight studies involving 2,049 patients investigated the correlation between the expression of LAG3 and RFS [27, 31, 35, 36, 53, 55, 59, 65]. The pooled analysis indicated no significant correlation between LAG3 expression and RFS (HR = 0.98, 95% CI 0.81–1.19, P = 0.871), as shown in Fig. 5. Subgroup analysis based on tumor type indicated that higher expression of LAG3 was correlated with worse RFS in patients with ESCA (HR = 1.72, 95% CI 1.06–2.79, P = 0.028). However, increased expression of LAG3 was associated with better RFS in patients with BRCA (HR = 0.87, 95% CI 0.78–0.97, P = 0.01) and OV (HR = 0.50, 95% CI 0.29–0.85, P = 0.01). No significant prognostic value of LAG3 expression was found in the RFS for BLCA, NSCLC, THCA, and UCEC (P > 0.05). Additionally, there was no significant correlation between LAG3 expression in TCs or TILs and RFS (P > 0.05) (Table 2 and Additional file 4: Figure S4).

#### Sensitive analysis and publication bias

We conducted a sensitivity analysis by sequentially excluding individual studies. For each component analysis, none of the HRs based on the remaining studies exceeded the estimated range. There was also no significant change observed between the adjusted pooled estimates and the main aggregate estimates, as depicted in Additional file 5: Figure S5. The robustness of our meta-analyses was thus confirmed. In addition, no publication bias was detected by funnel plots (Additional file 6: Figure S6).

### Pan-cancer analysis

#### Differential expression of LAG3 between normal and tumor samples

**Fig. 6:**
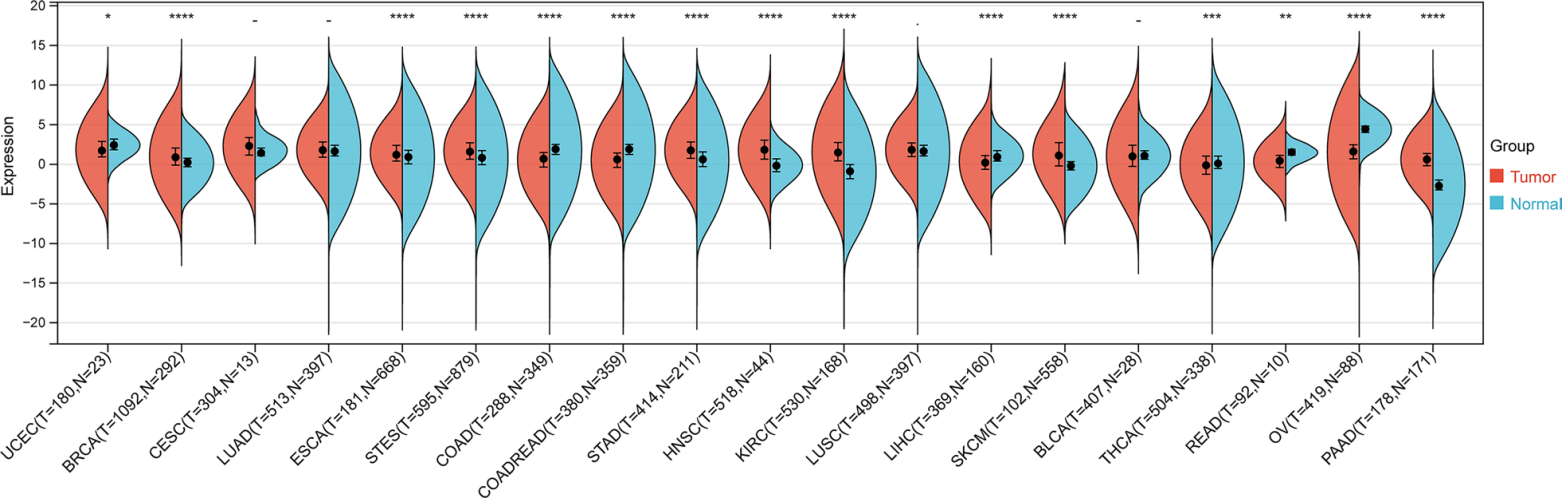
Transcriptomic expression differences of LAG3 between tumor and normal tissues in 19 kinds of cancers. *P < 0.05, **P < 0.01, ***P < 0.001, ****P < 0.0001

LAG3 expression was significantly upregulated in 8 tumors investigated in this study, including BRCA (tumor: 1.00 ± 1.60, normal: 0.20 ± 0.88, P = 1.1e-16), ESCA (tumor: 1.44 ± 1.51, normal: 0.85 ± 1.43, P = 5.6e-5), STES (tumor: 1.70 ± 1.51, normal: 0.78 ± 1.48, P = 5.4e-27), STAD (tumor: 1.81 ± 1.50, normal: 0.56 ± 1.63, P = 2.0e-19), HNSC (tumor: 1.80 ± 1.72, normal: – 0.11 ± 1.15, P = 3.0e-12), KIRC (tumor: 1.59 ± 1.86, normal: -0.96 ± 1.58, P = 2.7e-47), SKCM (tumor: 1.32 ± 2.15, normal: -0.22 ± 0.90, P = 1.8e-13), PAAD (tumor: 0.61 ± 1.22, normal: – 2.59 ± 1.50, P = 1.0e-48). However, the expression of LAG3 was significant downregulated in 7 tumors, including UCEC (tumor: 1.85 ± 1.66, normal: 2.42 ± 0.96, P = 0.04), COAD (tumor: 0.58 ± 1.55, normal: 1.73 ± 1.56, P = 1.7e-30), COADREAD (tumor: 0.53 ± 1.48, normal: 1.72 ± 1.54, P = 4.3e-39), LIHC (tumor: 0.39 ± 1.61, normal: 1.07 ± 1.03, P = 5.1e-11), THCA (tumor: – 0.05 ± 1.68, normal: 0.29 ± 1.56, P = 6.6e-4), READ (tumor: 0.36 ± 1.23, normal: 1.47 ± 0.71, P = 3.4e-3), OV (tumor: 1.53 ± 1.53, normal: 4.36 ± 0.76, P = 4.1e-42). No significant difference was observed between cancer and normal samples in BLCA, CESE, LUAD, and LUSC (P > 0.05) (Fig. 6).

#### Influence of CNV and SNV on the expression of LAG3

**Fig. 7:**
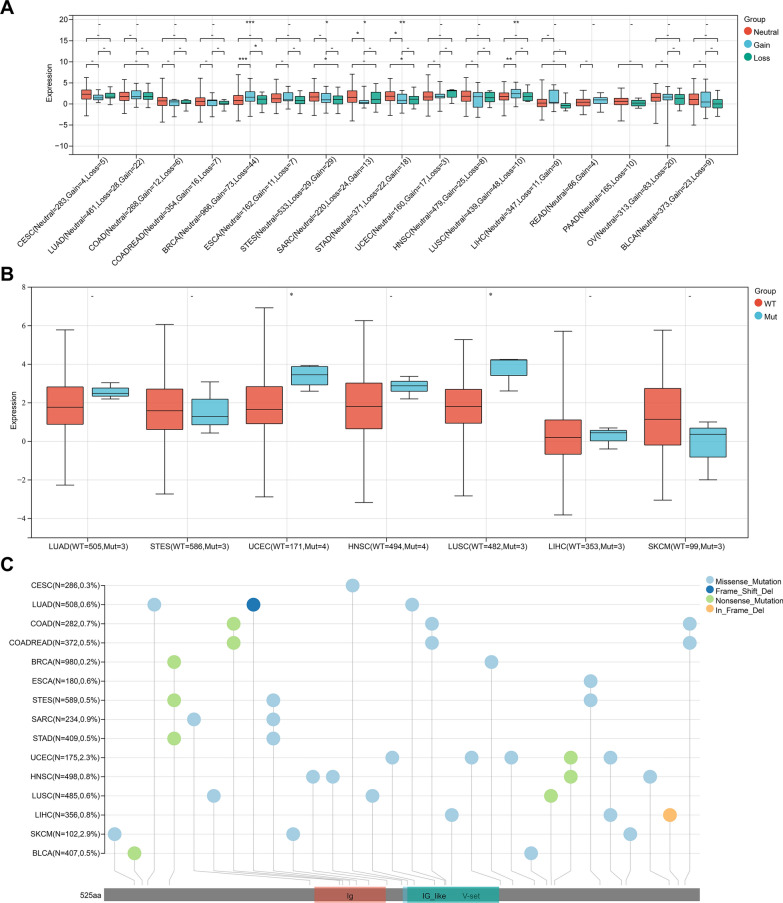
Genetic alteration of LAG3 in pan-cancer. **A** Correlation between LAG3 expression and copy number variation (CNV). **B** Correlation between LAG3 expression and single nucleotide variant (SNV). **C** Mutation diagram of LAG3 in different tumor types across protein domains. *P < 0.05, **P < 0.01, ***P < 0.001, ****P < 0.0001

The expression level of LAG3 in patients with CNV and SNV, and mutation landscape of LAG3 in different tumors across protein domains are presented in Fig. 7. LAG3 expression was significantly upregulated in CNV neutrals than in CNV gains in SARC and STAD. However, LAG3 expression was higher in CNV gains than in CNV neutrals in BRAC and LUSC. Additionally, LAG3 expression was significantly upregulated in CNV neutrals and CNV losses in STAD and STES. As for SNV, the expression of LAG3 was increased in SNV mutation than in SNV wild type in LUSC and UCEC. Missense mutations were found to be the most common type among the included tumors. In-frame deletion only occurred in LIHC. Moreover, SKCM presented the highest mutation frequency (2.9%).

#### RNA methylation modification-related gene analysis

**Fig. 8:**
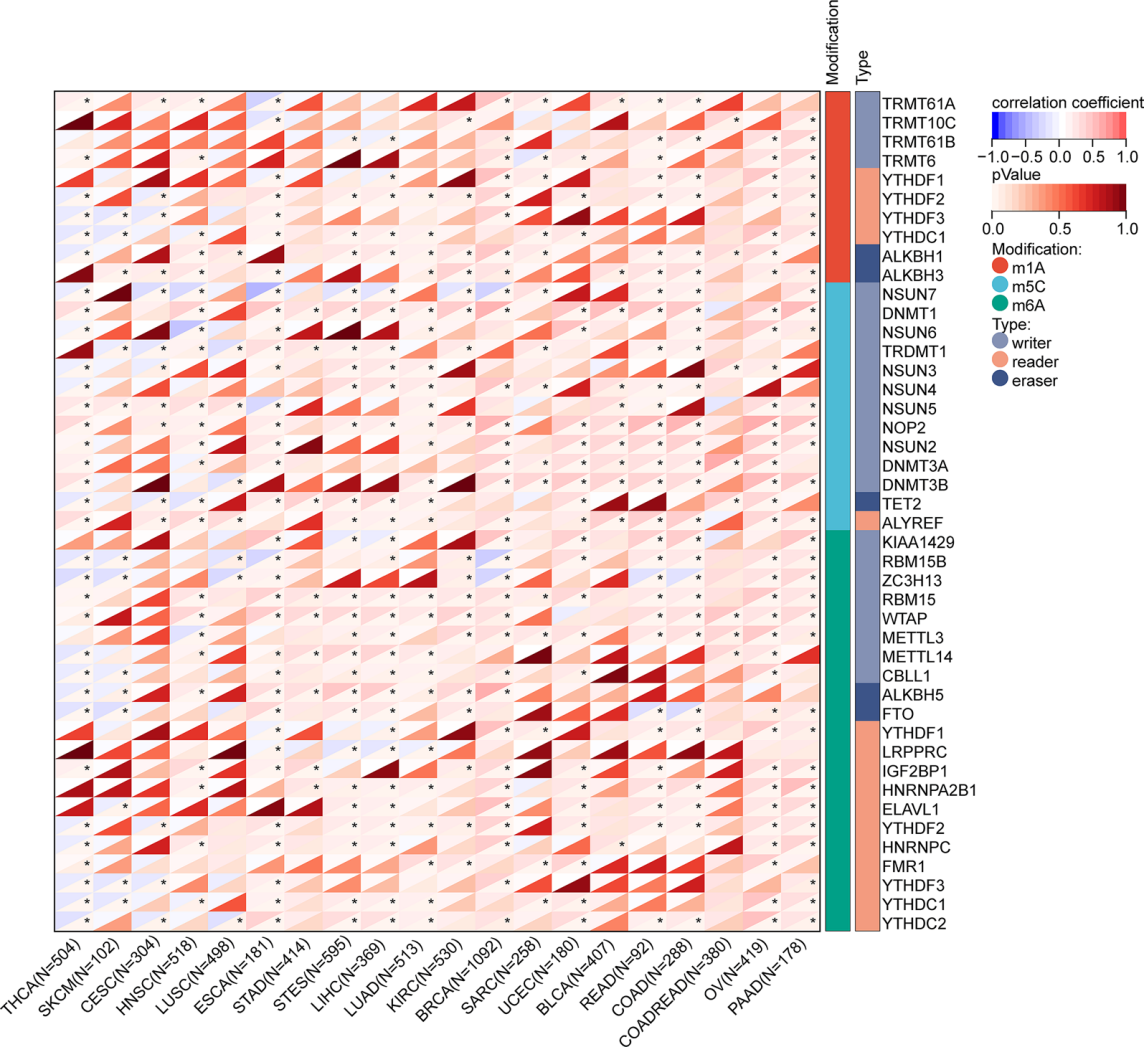
Spearman correlation of LAG3 expression with RNA methylation modifications-related gene including m1A, m5C and m6A in pan-cancer. *P < 0.05, **P < 0.01, ***P < 0.001, ****P < 0.0001

The correlation between LAG3 and RNA methylation modification-related genes is illustrated in Fig. 8. In the majority of tumors, positive associations were observed between the expression of LAG3 and most genes related to RNA methylation modifications. However, in certain tumor types, such as THCA, predominantly negative associations were identified. Briefly, our analysis indicated that the expression of LAG3 is likely to have significant impacts on RNA methylation modifications within tumors.

#### Relationship between LAG3 expression and TMB, MSI and NEO

**Fig. 9:**
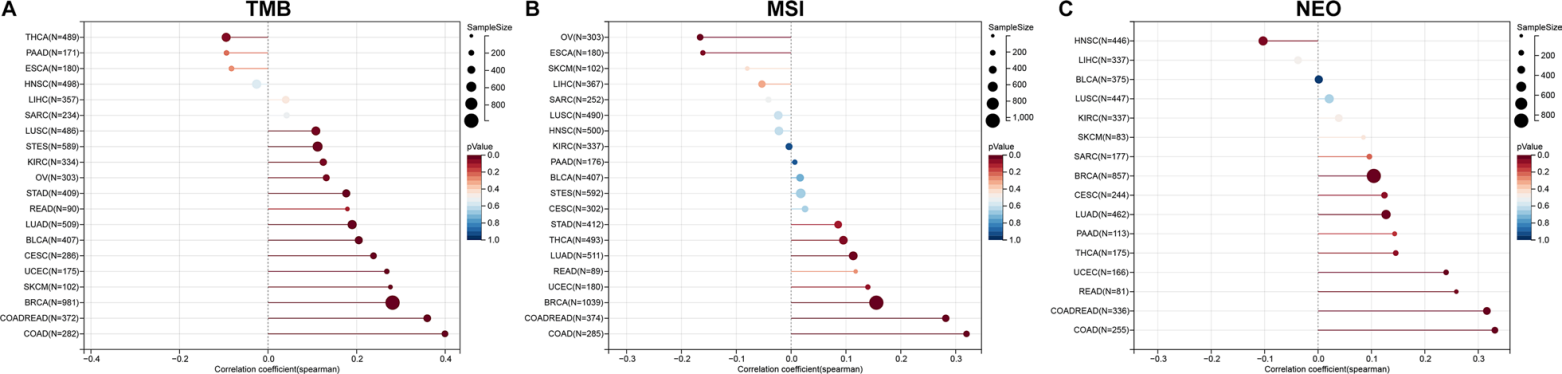
Correlation between LAG3 expression with genomic instability in pan-cancer. **A** Spearman correlation between LAG3 expression and TMB. **B** Spearman correlation between LAG3 expression and MSI. **C** Spearman correlation between LAG3 expression and NEO. TMB, tumor mutation burden; MSI, microsatellite instability; NEO, neoantigen

Given the crucial roles that TMB, MSI, and NEO play in predicting the response to immune therapy, we conducted Spearman correlation analyses to examine the associations between LAG3 expression and these factors. In BRCA, COAD, COADREA, LUAD, PAAD, READ, SARC, and UCEC, the expression of LAG3 was found to be positively correlated with TMB (Fig. 9A). In addition, LAG3 expression was negatively related to MSI in ESCA and OV, but positively related to MSI in BRCA, COAD, COADREAD, LUAD, and THCA (Fig. 9B). Moreover, the expression of LAG3 was positively associated with NEO in BRCA, COAD, COADREAD, LUAD, PAAD, READ, SARC, and UCEC (Fig. 9C). The results provided reliable evidence of a significant correlation between LAG3 and tumor immunity.

#### Immune infiltration analysis

**Fig. 10:**
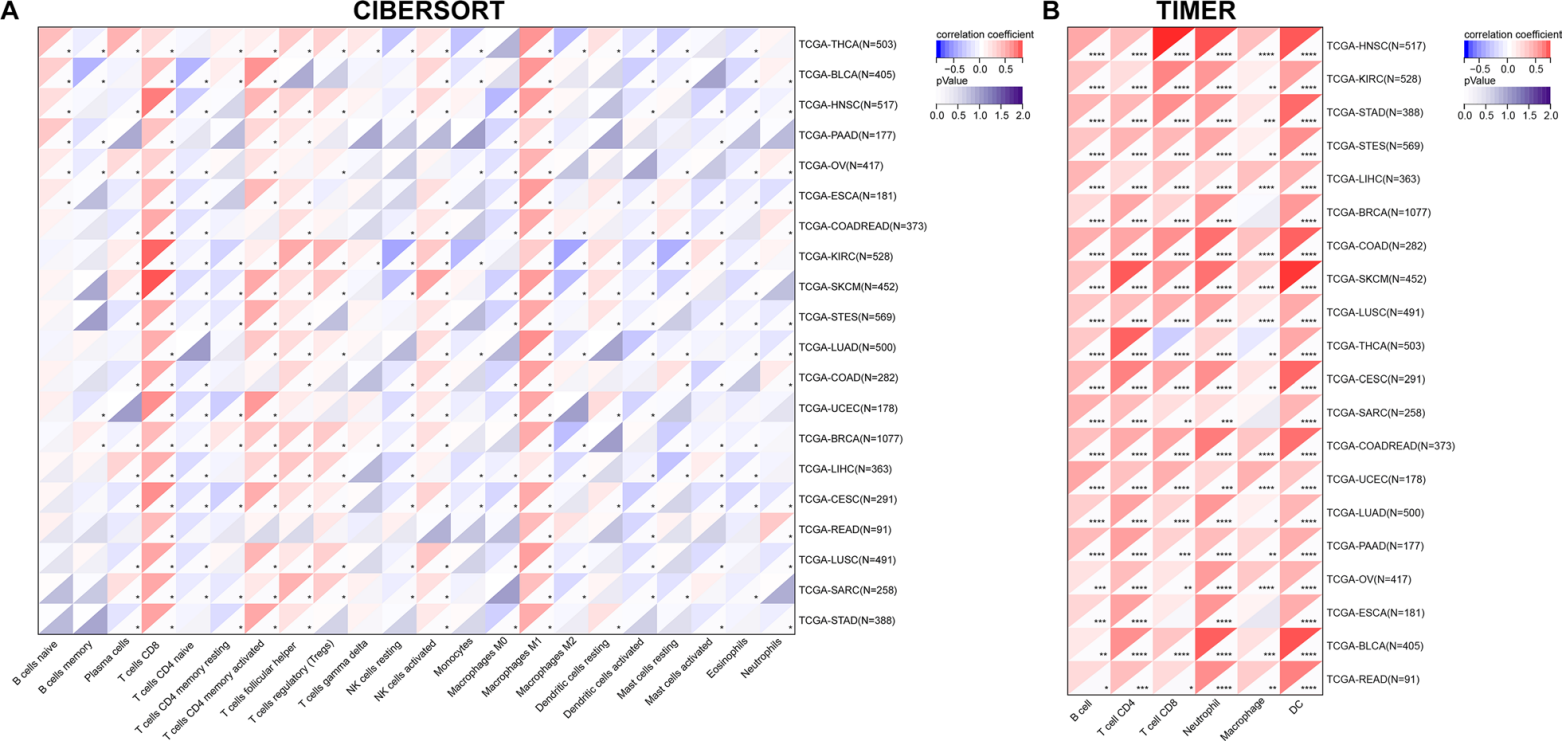
Correlation between LAG3 expression and immune cell infiltration in pan-cancer by the **A** CIBERSORT and **B** TIMER. *P < 0.05, **P < 0.01; ***P < 0.001; ****P < 0.0001

The correlation between LAG3 expression and immune cell infiltration was evaluated using two algorithms (CIBERSORT and TIMER). As shown in Fig. 10A, the expression of LAG3 was positively correlated with the infiltrating score of M1 macrophage and CD8^+^ T cells in all of tumor types we analyzed. LAG3 expression was negatively associated with activated dendritic cells (DC), activated mast cells, and eosinophils in most of the cancers included. Moreover, the expression of LAG3 was observed to have a positive correlation with the infiltration of B cells, CD4^+^ and CD8^+^ T cells, neutrophils, macrophages, and DC in the majority of tumors, as determined by the TIMER algorithm (Fig. 10B).

**Fig. 11:**
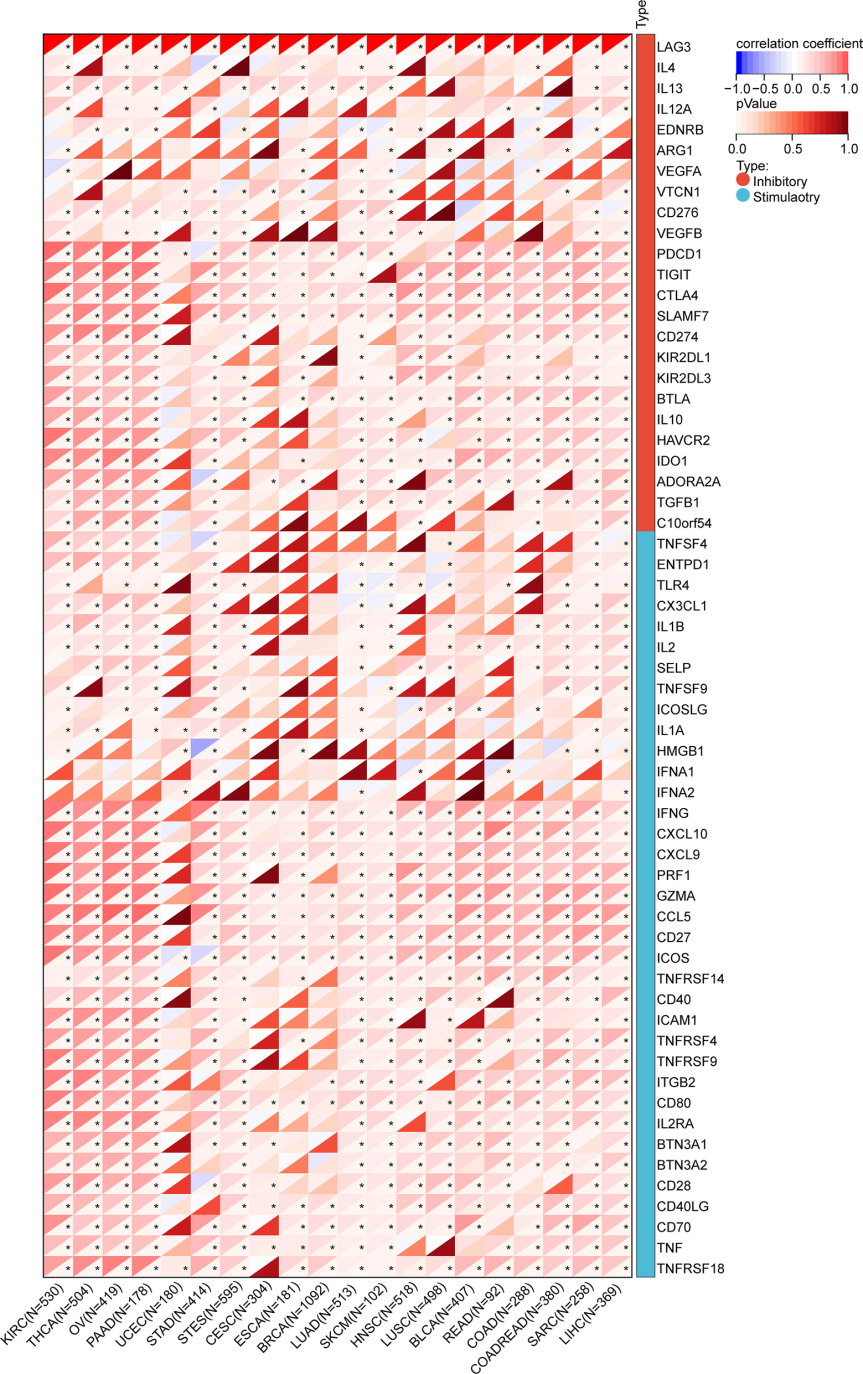
Spearman correlation between LAG3 expression and 60 immune checkpoint genes in pan-cancer. *P < 0.05, **P < 0.01; ***P < 0.001; ****P < 0.0001

Additionally, we investigated the relationship between LAG3 and 60 immune checkpoint genes using Spearman correlation analysis. The results demonstrated a significant correlation between LAG3 and a majority of immune-inhibitors and immune-stimulators, indicating a significant co-expression relationship. Notably, LAG3 expression demonstrated a positive association with the expression of several immune regulatory proteins, including programmed cell death protein 1 (PDCD1), CTLA-4, T-cell immunoglobulin and immunoreceptor tyrosine-based inhibitory motif domain (TIGIT), hepatitis A virus cellular receptor 2 (HAVCR2), interleukin 10 (IL-10), indoleamine 2,3-dioxygenase 1 (IDO1), B- and T-lymphocyte attenuator (BTLA), and transforming growth factor beta 1 (TGFB1) in the majority of tumors ( Fig. 11).

#### Construction of PPI network and enrichment analysis

**Fig. 12:**
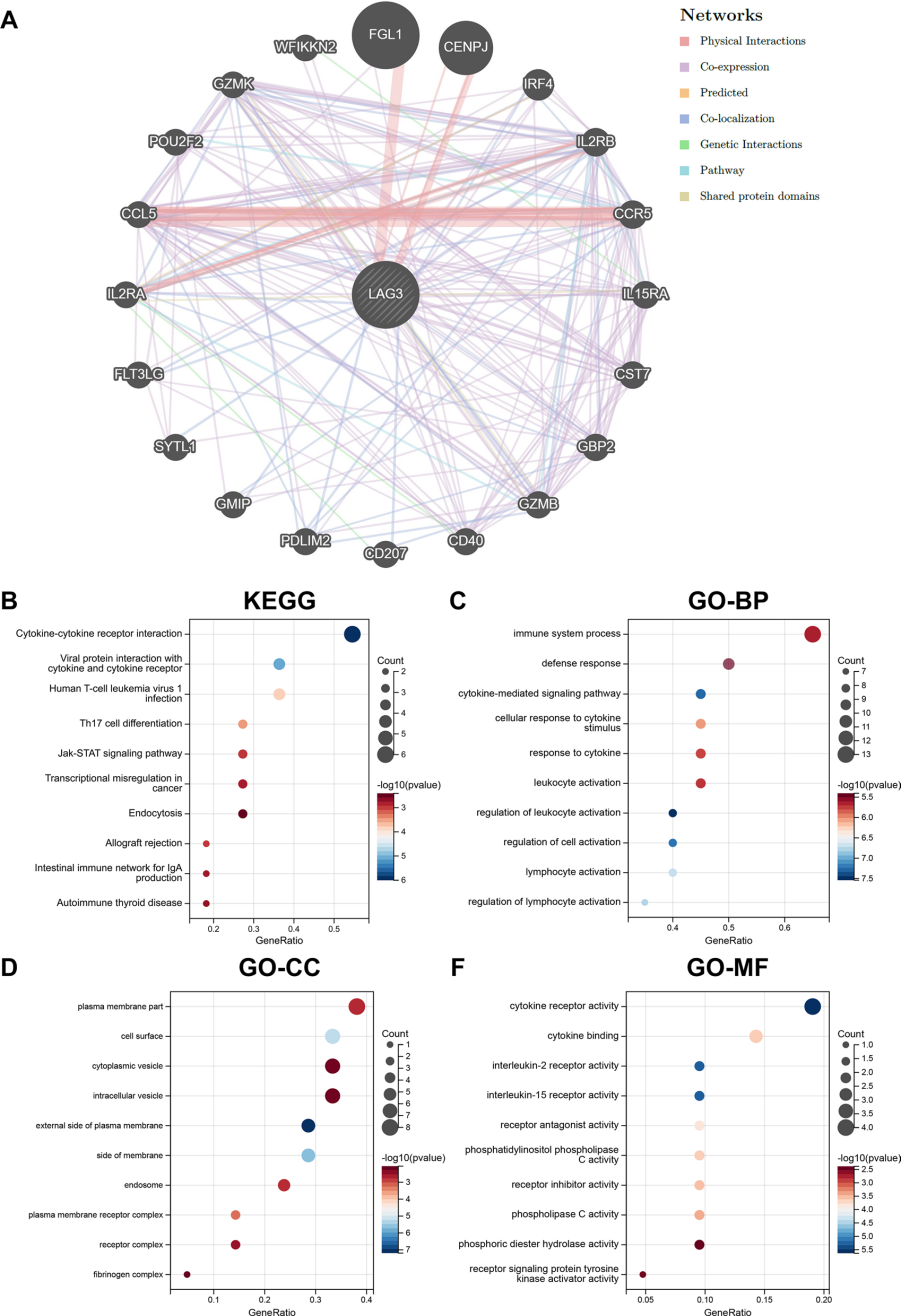
Visualization and enrichment analysis for genes that interacted with LAG3. **A** PPI network. **B** KEGG enrichment analysis. **C** GO-BP analysis. **D** GO-CC analysis. **E** GO-MF analysis. PPI, protein–protein interaction; KEGG, Kyoto Encyclopedia of Genes and Genomes; GO, Gene Ontology; BP, biological process; CC, cellular component; MF, molecular function

We used the GeneMANIA online program to construct a protein–protein interaction (PPI) network of the top 20 genes that interacted with LAG3, which is illustrated in Fig. 12A. The results of KEGG analysis showed that genes were mainly enriched in cytokine-cytokine receptor interaction, viral protein interaction with cytokine and cytokine receptor, human T-cell leukemia virus 1 infection, Th17 cell differentiation, and Jak-STAT signaling pathway (Fig. 12B). Genes in the GO analysis were most enriched in the BP of regulation of leukocyte activation (Fig. 12C), the CC of external side of plasma membrane (Fig. 12D), and the MF of cytokine receptor activity (Fig. 12F).

## Discussion

In recent years, there has been a growing body of literature examining the relationship between LAG3 expression and survival outcomes in different types of cancer. However, the prognostic significance of LAG3 in patients with solid tumors remains a topic of debate. In the present meta-analysis, we analyzed the data of 43 studies with 7,118 patients and quantitatively investigated the prognostic value of LAG3 in patients with solid tumors. The pooled analysis results revealed a significant association between higher expression of LAG3 and worse OS, but no significant associations with DFS, PFS or RFS. Subgroup analysis showed that LAG3 might play different prognostic roles in different solid tumors. Furthermore, pan-cancer bioinformatic analysis revealed that LAG3 was associated with genetic alterations, RNA methylation modification-related genes, genomic instability, immune checkpoint genes, and infiltration of immune cells. The results of this study suggested that LAG3 might be a potential prognostic marker and cancer immunotherapy target.

The LAG3 gene is composed of eight exons and is located on chromosome 12, adjacent to the CD4 gene, exhibiting a comparable intron–exon organization [6]. LAG3, as a co-inhibitory receptor, is primarily expressed on the surface of activated T cells (CD4^+^T cells and CD8^+^T cells), NK cells, B cells, and DCs in physiological conditions, and its primary role is to exert negative regulation on T cell function [67]. The ligands of LAG3 include major histocompatibility complex (MHC) class II molecules, fibrinogen-like protein 1 (FGL-1), alpha-synuclein (α-syn), galectin 3 (Gal-3), and LSECtin [68–71]. As an immune checkpoint, LAG3 inhibits the activation of host immune cells and suppresses immune responses [72, 73]. Previous studies have provided evidence suggesting that LAG3 acts as an immune checkpoint, conferring immunosuppressive properties and facilitating tumor progression within the TME. For example, by binding to its ligands and mediation of inhibitory cytokines secretion by Tregs, LAG3 can negatively regulate the activation, proliferation, effector function, and homeostasis of both CD8^+^ and CD4^+^ T cells [63, 74, 75]. LAG3 also exhibits a synergistic effect with PD-1/PD-L1 in the suppression of antitumor immune responses [76, 77]. Consequently, this indicates a negative prognostic value of LAG3 across different cancer types [29, 63]. It has been reported that the concurrent administration of LAG3 inhibitors alongside anti-PD-1 or PD-L1 agents can result in an enhanced effect [78, 79]. Moreover, current clinical investigations are being conducted to assess the efficacy of these combinations. To develop novel molecule-targeted immunotherapy, more studies are required to determine the biochemical and molecular pathways through which LAG3 influences immune responses and tumor growth.

Due to its significant involvement in the formation of the tumor immunosuppressive microenvironment, LAG3 is anticipated to be correlated with an unfavorable prognosis in cancer patients. However, a previously published meta-analysis showed that the overexpression of LAG3 was correlated with a more favorable OS in several types of cancer, which could be attributed to the limited number of included studies and the enrollment of a large number of patients with early-stage cancer [80]. In contrast to prior research, we found that higher expression of LAG3 was associated with worse OS in patients with solid tumors. The inclusion of 43 studies involving 18 types of solid tumors increases the confidence of our results to some extent. Subgroup analyses based on tumor type indicated that higher LAG3 expression was significantly correlated with worse survival outcomes in BLCA, CESE, ESCA, KIRC, LIHC, PAAD, SARC, and SKCM. Interestingly, we found that higher expression of LAG3 was associated with better DFS of COAD, RFS of BRAC and OV. Similarly, Hu et al. reported that the infiltration of LAG3^+^ lymphocytes ameliorated OS in patients with triple-negative breast cancer [81]. Therefore, LAG3 might potentially serve as a favorable prognostic biomarker for BRCA. Considering the limited number of included studies, it is essential to conduct further investigations in future research studies to explore the prognostic significance of LAG3 in COAD and OV. Although it is indeed possible that specific similarities or tumor traits that influence how LAG3 influences patient outcomes, we could not identify them at present due to the limited number of studies. In addition, subgroup analyses based on the location of LAG3 expression showed that higher expression of LAG3 in both TCs and TILs was associated with poorer survival outcomes, suggesting LAG3 has the potential to be used as a prognostic biomarker for patients with solid tumors, regardless of its expression location.

It is worth noting that combining LAG3 with other biomarkers may improve prognosis and treatment response prediction in patients with cancer. In LIHC, the co-expression of FGL-1 and LAG3 is inversely associated with the number of CD8^+^T cells, which predict a poor survival [34]. In gastric cancer, the co-expression of PD-1 and LAG3 is indicative of improved PFS, and the co-expression of TIM3 and LAG3 is associated with better OS and PFS [47]. Additionally, LAG3 combined with PD-1/PD-L1 may serve as a predictor of immunotherapy treatment effectiveness in primary pulmonary lymphoepithelioma-like carcinoma [82]. However, the specific molecular and biological mechanisms through which LAG3 and PD-1/PD-L1 act synergistically have not yet been elucidated. Future research is supposed to further investigate the possible synergy between LAG3 and other biomarkers in predicting cancer prognosis and treatment response. However, there is still some way from the clinical application of LAG3 as a prognostic biomarker because the role of LAG3 in prognosis prediction is controversial in different solid tumors. Further multicenter prospective randomized controlled trials with larger sample sizes are required before its clinical application.

Tumor-infiltrating immune cells have the ability to either facilitate or hinder the development and progression of tumors [83]. The consistent association of LAG3 expression levels and the infiltration of LAG3^+^ immune cells within the TME with tumor progression and unfavorable prognosis across diverse human tumor types strongly indicates the involvement of LAG3 in a PD-1-like tumor immune escape mechanism [77]. LAG3 can exhaust activated T cells and up-regulate the function of Tregs to reduce the immune response [72]. Furthermore, LAG3 has the capability to suppress the proliferation and activity of T cells and NK cells through its mediation of IL-10 and IL-35 secretion by Treg [84]. In this study, we found that high LAG3 expression increased the infiltration levels of CD8^+^ T cells, M1 macrophage, Tregs, activated memory CD4^+^ T cells, follicular helper T cell, and activated NK cells in most of the solid tumors included. Moreover, a noteworthy correlation was identified between LAG3 expression and a majority of other immune checkpoints, such as PDCD1, CTLA-4, TIGIT, and HAVCR2. The results suggested that LAG3 might play a pivotal role in various immune responses and the infiltration of immune cells. Consequently, the simultaneous inhibition of LAG3 and other immune checkpoints could potentially be considered as a novel approach for immunotherapy. The TMB, MSI, and NEO are three significant indicators for predicting the sensitivity and therapeutic effect of immune checkpoint inhibitors [85, 86]. In this study, we found a positive correlation between LAG3 expression and TMB, MSI, and NEO in BRCA, COAD, COADREAD, and LUAD, suggesting that patients with these tumors may potentially benefit from LAG3 inhibitors. Consequently, LAG3 emerges as a potentially valuable biomarker for predicting the efficacy of immunotherapy.

Previous studies have reported that RNA methylation could influence LAG3's function in tumor immunity. For example, tumor-intrinsic protein and LAG3 mRNA expression are linked to methylation regulation in KIRC. The hypomethylation of the LAG3 promoter and the methylation of LAG3 downstream genes may be related to the overexpression of LAG3 mRNA. Additionally, the hypomethylation of LAG3 promoter and CpG site 15 have been found to be associated with increased infiltration of immune cells [87]. In melanoma, higher LAG3 mRNA expression is linked to the hypomethylation of beads 1 through 13, and the hypomethylation of the LAG3 promoter and the CTCF binding site may enhance immune cell infiltration [88]. In this study, we found that the expression of LAG3 was closely associated with RNA methylation modification-related genes in most solid tumors, indicating that the methylation of LAG3 has the potential to serve as a novel epigenetic biomarker for the infiltration of immune cells in tumors. Future studies are supposed to further explore this issue.

Currently, there is a need to identify novel immune checkpoints that can effectively address the challenges of drug resistance and the occurrence of severe adverse reactions linked to PD-1/PD-L1 and CTLA-4 inhibitors [5]. In light of the positive findings from studies on LAG3, a number of LAG3 inhibitors, including relatlimab, eftilagimod alpha, RO7247669, SHR-1802, GSK2831781 have been under clinical trials in patients with various solid tumors [89–91]. Due to the co-expression of LAG3 and PD-1/PD-L1, LAG3 inhibitors are currently used more frequently with PD-1/PD-L1 inhibitors. RELATIVITY-047, a global, randomized, double-blind Phase 2/3 study of patients with metastatic or unresectable melanoma in the first-line setting, recently demonstrated the clinical success of an anti-LAG3/PD-1 combination therapy and led to the FDA approving relatlimab with nivolumab. The anti-LAG3/PD-1 combination therapy demonstrated a significant improvement in PFS compared to nivolumab monotherapy (10.2 months vs. 4.6 months). In addition, patients with LAG3 expression greater than 1% did show improved PFS with the addition of relatlimab to nivolumab [89]. The anti-LAG3/PD-1 combination therapy experienced 21.1% of grade 3 or 4 adverse events, which were controllable. This safety profile was significantly better than that of anti-PD-1/CTLA-4 combination treatment, where 59% of patients experienced Grade 3 or 4 toxicity [92]. The utilization of LAG3 inhibitors holds promise as a potential immunotherapy in the future. However, there are still few studies on the resistance mechanisms and negative effects of LAG3-targeted therapy. Further research investigating potential negative effects and resistance mechanisms, as well as the feasibility and efficacy of LAG3-targeted medicines should be conducted in the nearly future.

This study has several limitations that should be acknowledged. Firstly, all the included studies were retrospective cohort studies, which are prone to inherent biases like cohort selection bias, potentially compromising the reliability of the findings. Secondly, the cut-off values for LAG3 expression and the method used to determine these values varied among the included studies, possibly leading to a selection bias and heterogeneity of the results. Thirdly, some of the included studies had a limited scale. Additionally, certain aspects of the subgroup analysis were based on a relatively small number of studies, potentially leading to bias and reduced confidence in results. Finally, differences in tumor stage and treatment methods adopted by the patients enrolled in the studies included in the analysis contributed to some extent to the differences in the subgroup analyses. Given these limitations, further multicenter prospective randomized controlled trials with larger sample sizes are necessary to validate the results before widespread implementation in clinical practice.

## Conclusion

LAG3 has a substantial prognostic value in patients diagnosed with solid tumors. High expression of LAG3 is consistently associated with an unfavorable prognosis in solid cancer patients, and LAG3 might play different prognostic roles in different solid tumors. LAG3 exhibits correlations with genetic alterations, RNA methylation modification-related genes, immune cell infiltration, immune-related genes, TMB, MSI, and NEO. Consequently, LAG3 may serve as a novel prognostic biomarker for patients with solid tumors, and the use of LAG3 inhibitors holds promise as a potential therapeutic approach in the future.

### Supplementary Information

**Additional file 1: Figure S1.** Subgroup analysis of overall survival (OS). **A** Subgroup analysis based on tumor types. **B** Subgroup analysis based on LAG3 expression location.

**Additional file 2: Figure S2.** Subgroup analysis of disease-free survival (DFS). **A** Subgroup analysis based on tumor types. **B** Subgroup analysis based on LAG3 expression location.

**Additional file 3: Figure S3.** Subgroup analysis of progression-free survival (PFS). **A** Subgroup analysis based on tumor types. **B** Subgroup analysis based on LAG3 expression location.

**Additional file 4: Figure S4.** Subgroup analysis of recurrence-free survival (RFS). **A** Subgroup analysis based on tumor types. **B** Subgroup analysis based on LAG3 expression location.

**Additional file 5: Figure S5.** Sensitivity analysis of **A** overall survival, **B** disease-free survival, **C** progression-free survival, **D** recurrence-free survival.

**Additional file 6: Figure S6.** Publication bias detected by funnel plots of **A** overall survival, **B** disease-free survival, **C** progression-free survival, **D** recurrence-free survival.

**Additional file 7: Table S1a.** English literature retrieval strategy (Pubmed). **b** English literature retrieval strategy (EMBASE). **c** English literature retrieval strategy (Cochrane Library).

**Additional file 8: Table S2.** Detailed quality assessment of case-control studies.

## Data Availability

The data used and/or analysed during the current study are available from the corresponding author on reasonable request.

## Abbreviations

LAG3: Lymphocyte-activation gene 3
OS: Overall survival
DFS: Disease-free survival
PFS: Progression-free survival
RFS: Recurrence-free survival
HR: Hazard ratio
CI: Confidence interval
TME: Tumor microenvironment
PD-1: Programmed cell death 1
PD-L1: Programmed cell death ligand 1
CTLA-4: Cytotoxic T lymphocyte antigen 4
ICI: Immune checkpoint inhibitor
Treg: Regulatory T cell
NK: Natural killer
DC: Dendritic cells
PRISMA: Preferred Reporting Items for Systematic Reviews and Meta-Analyses
MOOSE: Meta-Analysis of Observational Studies in Epidemiology
MeSH: Medical Subject Headings
NOS: Newcastle–Ottawa Quality Assessment Scale
BLCA: Bladder urothelial carcinoma
BRCA: Breast invasive carcinoma
CESC: Cervical squamous cell carcinoma and endocervical adenocarcinoma
COAD: Colon adenocarcinoma
COADREAD: Colon adenocarcinoma and rectum adenocarcinoma
ESCA: Esophageal carcinoma
HNSC: Head and neck squamous cell carcinoma
KIRC: Kidney renal clear cell carcinoma
LIHC: Liver hepatocellular carcinoma
LUAD: Lung adenocarcinoma
LUSC: Lung squamous cell carcinoma
OV: Ovarian serous cystadenocarcinoma
PAAD: Pancreatic adenocarcinoma
READ: Rectum adenocarcinoma
SARC: Sarcoma
SKCM: Skin cutaneous melanoma
STAD: Stomach adenocarcinoma
STES: Stomach and esophageal carcinoma
THCA: Thyroid carcinoma
UCEC: Uterine corpus endometrial carcinoma
m1A: N1-methyladenosine
m5C: 5-Methylcytidine
m6A: N6-methyladenosine
SNV: Simple nucleoside variation
CNV: Copy number variation
TMB: Tumor mutation burden
MSI: Microsatellite instability
NEO: Neoantigen
PPI: Protein–protein interaction
GO: Gene Ontology
KEGG: Kyoto Encyclopedia of Genes and Genomes
BP: Biological process
CC: Cellular component
MF: Molecular function
TIL: Tumor-infiltrating lymphocyte
TC: Tumor cell
TAM: Tumor-associated macrophage
TIGIT: T-cell immunoglobulin and immunoreceptor tyrosine-based inhibitory motif domain
HAVCR2: Hepatitis A virus cellular receptor 2
IDO1: Indoleamine 2,3-dioxygenase 1
IL: Interleukin
BTLA: B- and T-lymphocyte attenuator
TGFB1: Transforming growth factor beta 1
MHC: Major histocompatibility complex
FGL-1: Fibrinogen-like protein 1
α-syn: Alpha-synuclein
Gal-3: Galectin 3
