# Supplementary figures and images for: Prognostic significance of Lymphocyte-activation gene 3 (LAG3) in patients with solid tumors: a systematic review, meta-analysis and pan-cancer analysis
Source: Cancer Cell Int. 2023 Dec 2;23:306. doi: 10.1186/s12935-023-03157-5 (PMC10693146; doi:10.1186/s12935-023-03157-5)

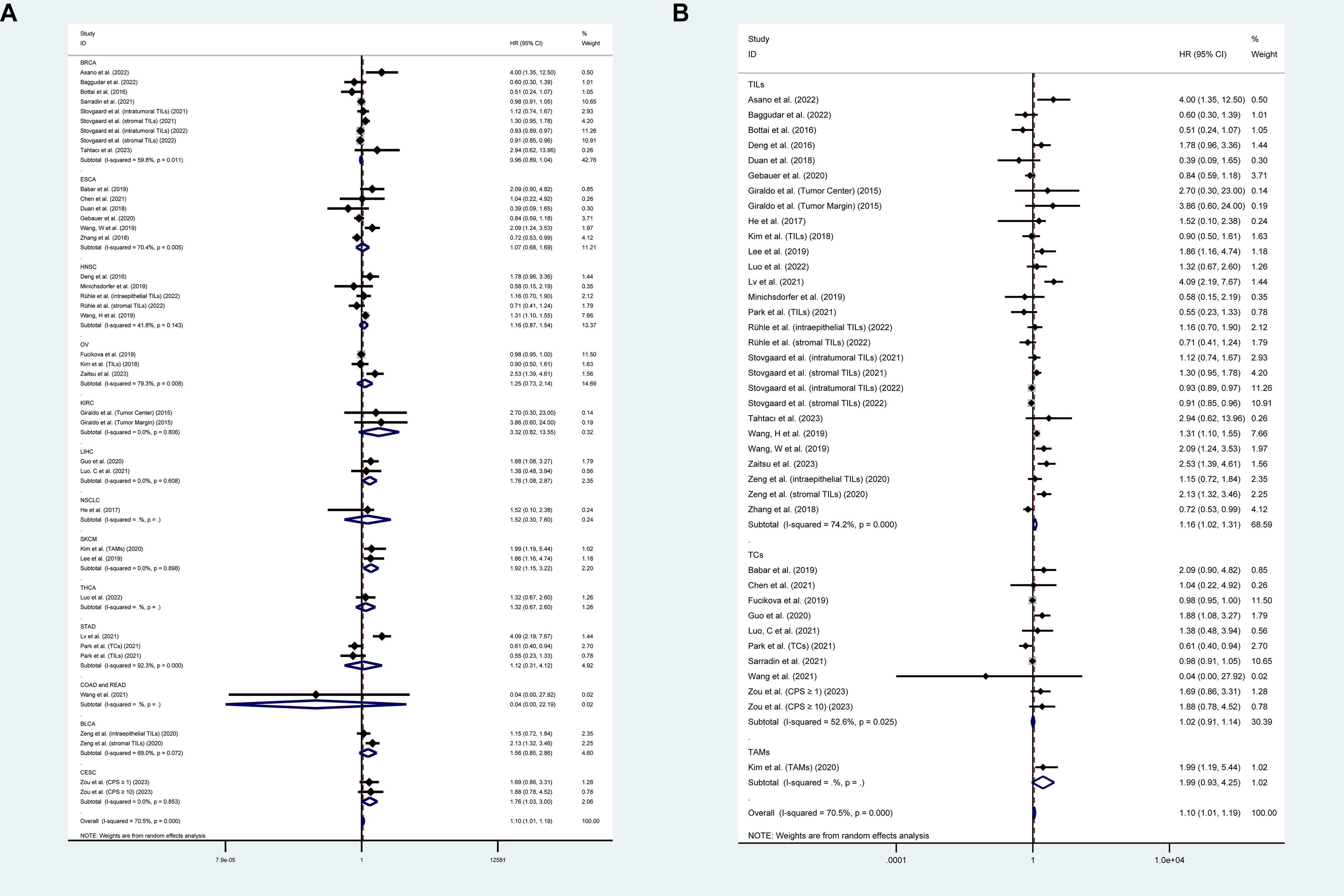

Supplement: Supplementary file 1 — Additional file 1: Figure S1. Subgroup analysis of overall survival (OS). A Subgroup analysis based on tumor types. B Subgroup analysis based on LAG3 expression location. [file 12935_2023_3157_MOESM1_ESM.tif]

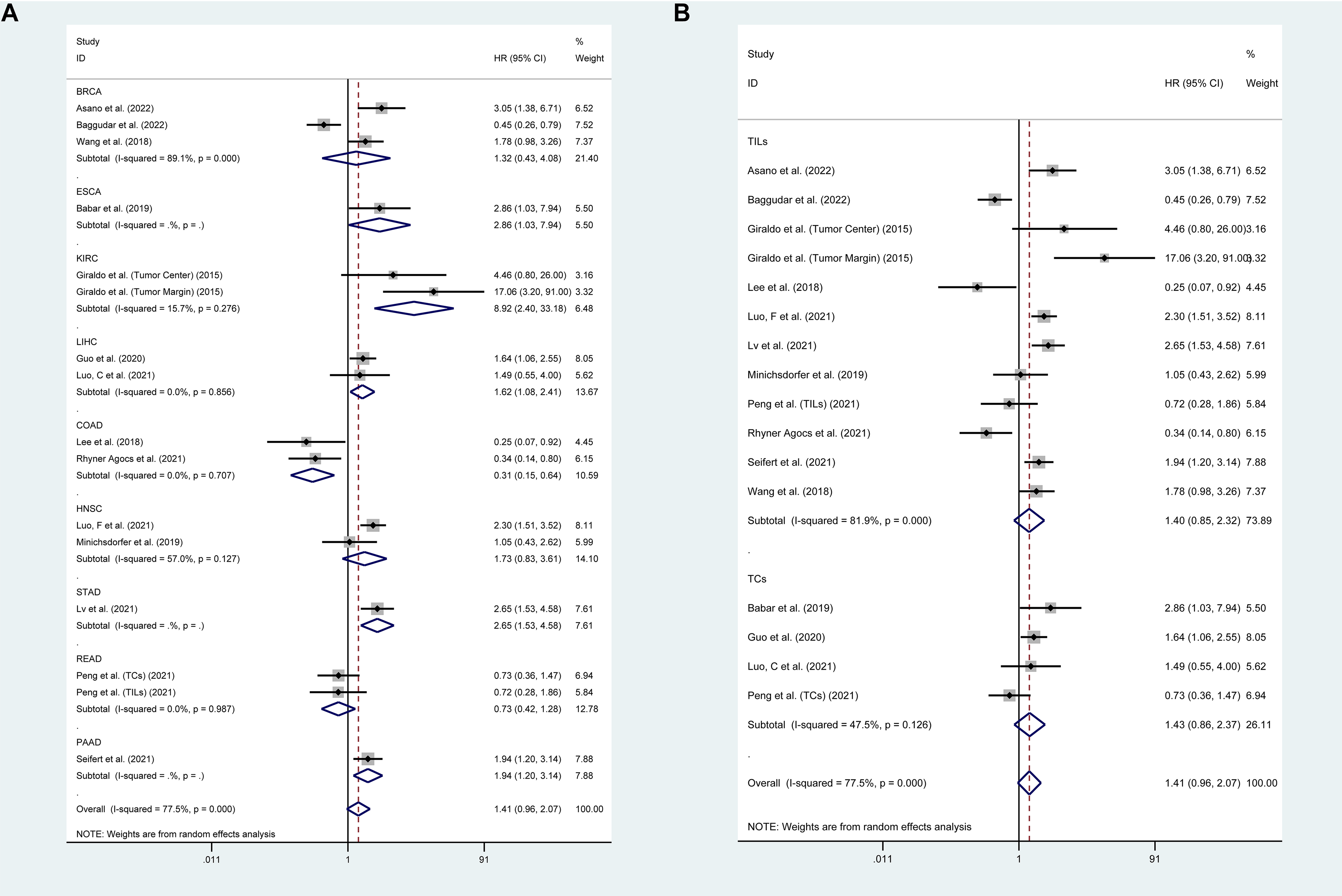

Supplement: Supplementary file 2 — Additional file 2: Figure S2. Subgroup analysis of disease-free survival (DFS). A Subgroup analysis based on tumor types. B Subgroup analysis based on LAG3 expression location. [file 12935_2023_3157_MOESM2_ESM.tif]

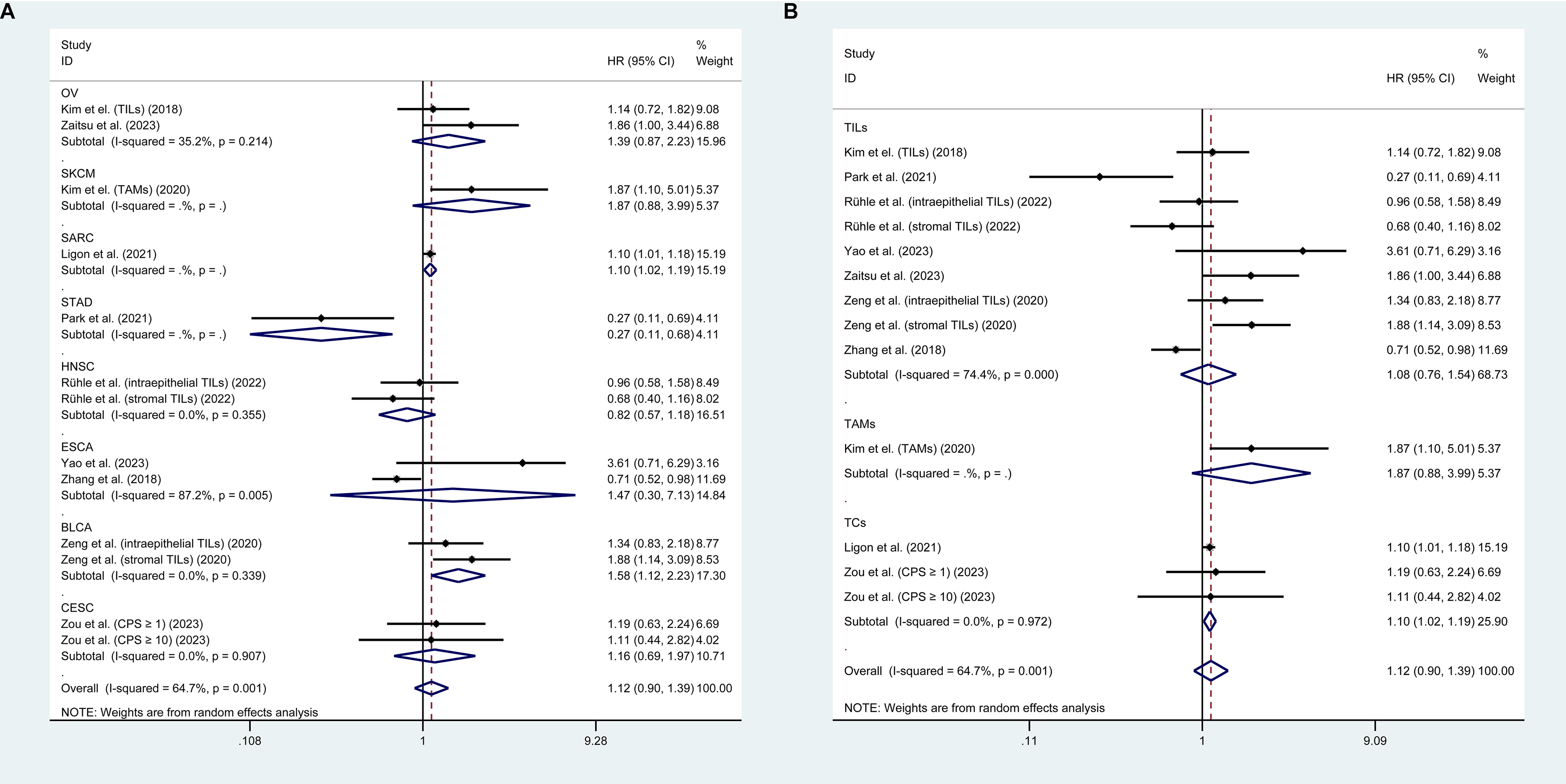

Supplement: Supplementary file 3 — Additional file 3: Figure S3. Subgroup analysis of progression-free survival (PFS). A Subgroup analysis based on tumor types. B Subgroup analysis based on LAG3 expression location. [file 12935_2023_3157_MOESM3_ESM.tif]

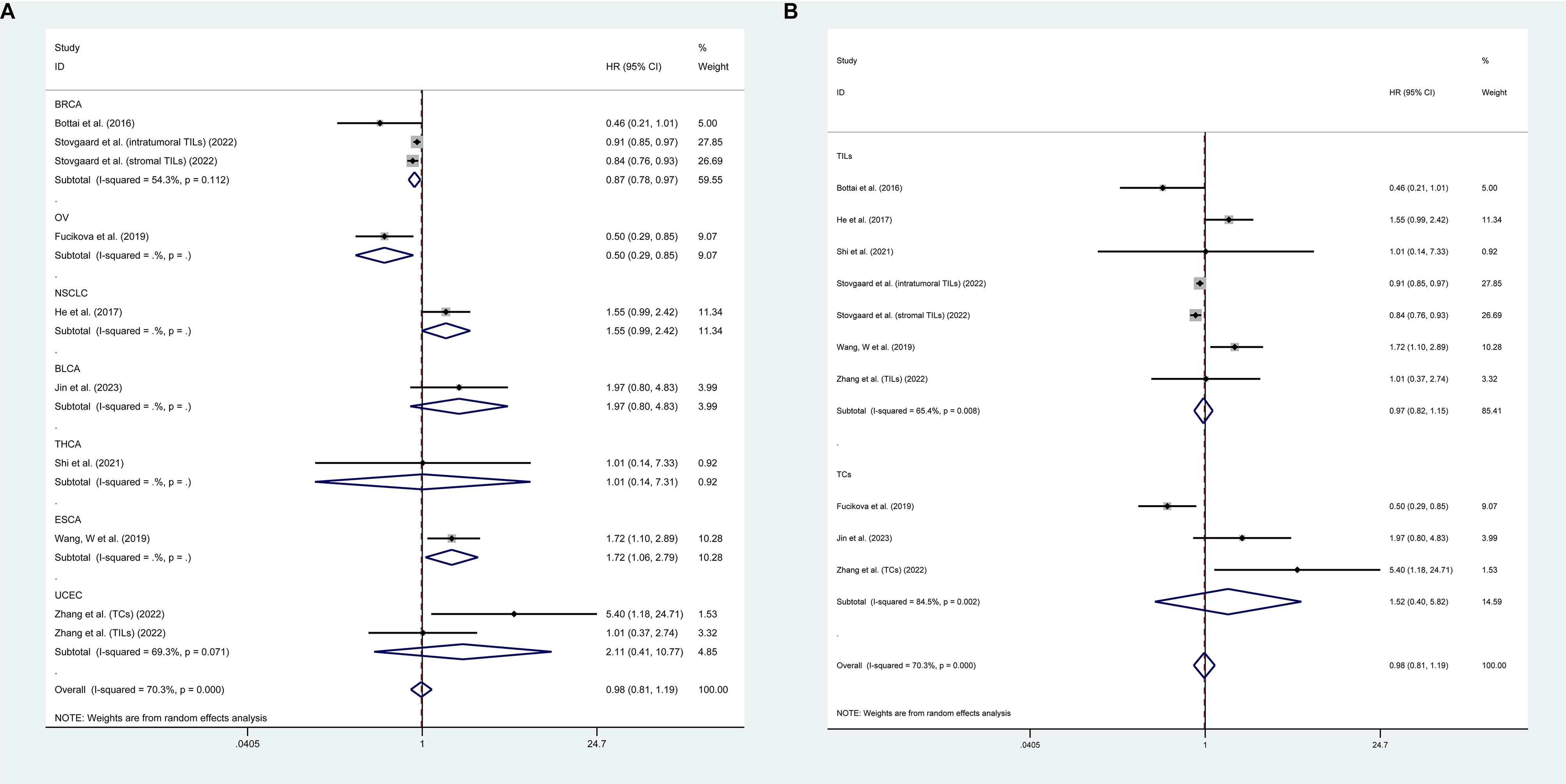

Supplement: Supplementary file 4 — Additional file 4: Figure S4. Subgroup analysis of recurrence-free survival (RFS). A Subgroup analysis based on tumor types. B Subgroup analysis based on LAG3 expression location. [file 12935_2023_3157_MOESM4_ESM.tif]

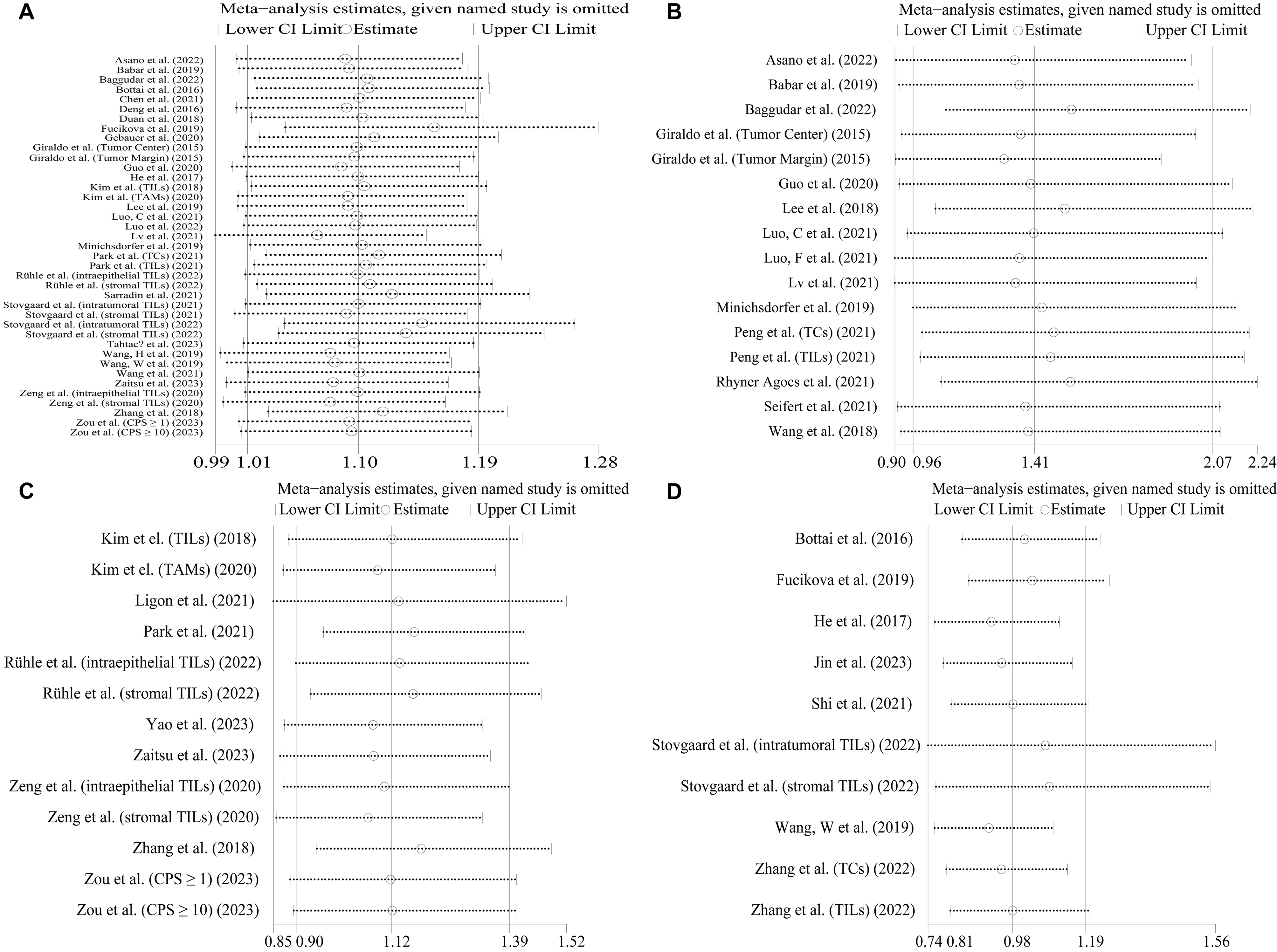

Supplement: Supplementary file 5 — Additional file 5: Figure S5. Sensitivity analysis of A overall survival, B disease-free survival, C progression-free survival, D recurrence-free survival. [file 12935_2023_3157_MOESM5_ESM.tif]

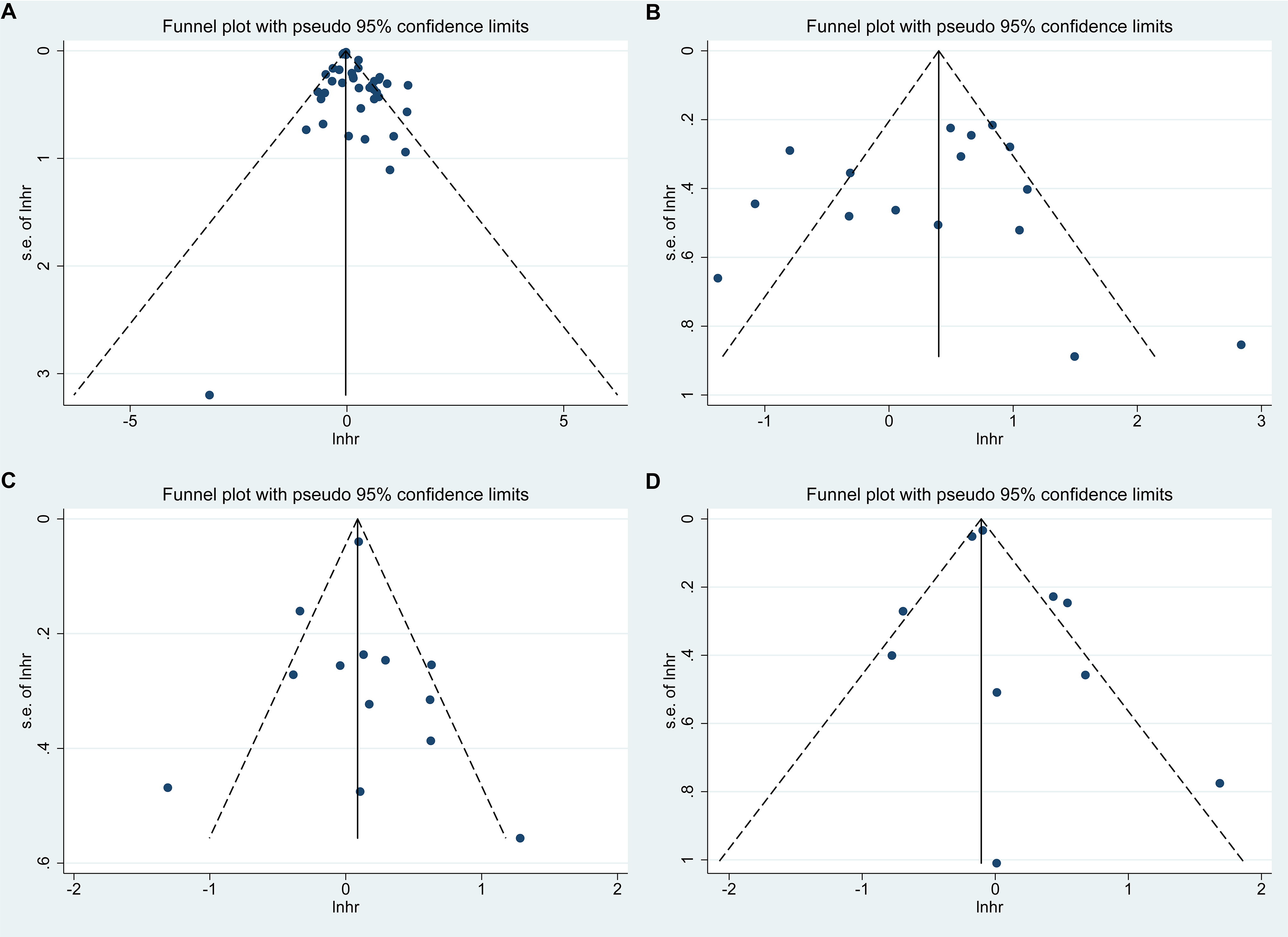

Supplement: Supplementary file 6 — Additional file 6: Figure S6. Publication bias detected by funnel plots of A overall survival, B disease-free survival, C progression-free survival, D recurrence-free survival. [file 12935_2023_3157_MOESM6_ESM.tif]
