# Supplementary material for: Prognostic significance of Lymphocyte-activation gene 3 (LAG3) in patients with solid tumors: a systematic review, meta-analysis and pan-cancer analysis
Source: Cancer Cell Int. 2023 Dec 2;23:306. doi: 10.1186/s12935-023-03157-5 (PMC10693146; doi:10.1186/s12935-023-03157-5)
Supplement: Supplementary file 7 — Additional file 7: Table S1a. English literature retrieval strategy (Pubmed). b English literature retrieval strategy (EMBASE). c English literature retrieval strategy (Cochrane Library). [file 12935_2023_3157_MOESM7_ESM.docx]

**Table S1a.** English literature retrieval strategy (Pubmed).

| **Number** | **Search terms** |
| --- | --- |
| 1 | LAG3 [Title/Abstract] |
| 2 | Lymphocyte activating 3 [Title/Abstract] |
| 3 | CD223 [Title/Abstract] |
| 4 | 1 OR 2 OR 3 |
| 5 | Carcinoma [MeSH] |
| 6 | Carcinoma [Title/Abstract] |
| 7 | Neoplasms [MeSH] |
| 8 | Neoplasms [Title/Abstract] |
| 9 | Cancer [Title/Abstract] |
| 10 | Neoplasia [Title/Abstract] |
| 11 | Tumor [Title/Abstract] |
| 12 | Malignancy [Title/Abstract] |
| 13 | Malignant neoplasm [All Fields] |
| 14 | 5 OR 6 OR 7 OR 8 OR 9 OR 10 OR 11 OR 12 OR 13 |
| 15 | Prognosis [MeSH] |
| 16 | Prognosis [Title/Abstract] |
| 17 | Survival analysis [MeSH] |
| 18 | Survival analysis [Title/Abstract] |
| 19 | Prognostic [Title/Abstract] |
| 20 | Treatment outcome [MeSH] |
| 21 | Treatment outcome [Title/Abstract] |
| 22 | Fatal outcome [MeSH] |
| 23 | Fatal outcome [Title/Abstract] |
| 24 | Mortality [MeSH] |
| 25 | Mortality [Title/Abstract] |
| 26 | 15 OR 16 OR 17 OR 18 OR 19 OR 20 OR 21 OR 22 OR 23 OR 24 OR 25 |
| 27 | 4 AND 14 AND 26 |

**Table S1b.** English literature retrieval strategy (EMBASE).

| **Number** | **Search terms** |
| --- | --- |
| 1 | LAG3 [Title/Abstract] |
| 2 | Lymphocyte activating 3 [Title/Abstract] |
| 3 | CD223 [Title/Abstract] |
| 4 | 1 OR 2 OR 3 |
| 5 | Carcinoma [Title/Abstract] |
| 6 | Neoplasms [Title/Abstract] |
| 7 | Cancer [Title/Abstract] |
| 8 | Neoplasia [Title/Abstract] |
| 9 | Tumor [Title/Abstract] |
| 10 | Malignancy [Title/Abstract] |
| 11 | Malignant neoplasm [Title/Abstract] |
| 12 | 5 OR 6 OR 7 OR 8 OR 9 OR 10 OR 11 |
| 13 | Prognos* [Title/Abstract] |
| 14 | Survival analysis [Title/Abstract] |
| 15 | Treatment outcome [Title/Abstract] |
| 16 | Fatal outcome [Title/Abstract] |
| 17 | Mortality [Title/Abstract] |
| 18 | 13 OR 14 OR 15 OR 16 OR 17 |
| 19 | 4 AND 12 AND 18 |

**Table S1c.** English literature retrieval strategy (Cochrane Library).

| **Number** | **Search terms** |
| --- | --- |
| 1 | LAG3 [Title Abstract Keyword] |
| 2 | Lymphocyte activating 3 [Title Abstract Keyword] |
| 3 | CD223 [Title Abstract Keyword] |
| 4 | 1 OR 2 OR 3 |
| 5 | Carcinoma [Title Abstract Keyword] |
| 6 | Neoplasms [Title Abstract Keyword] |
| 7 | Cancer [Title Abstract Keyword] |
| 8 | Neoplasia [Title Abstract Keyword] |
| 9 | Tumor [Title Abstract Keyword] |
| 10 | Malignancy [Title Abstract Keyword] |
| 11 | Malignant neoplasm [Title Abstract Keyword] |
| 12 | 5 OR 6 OR 7 OR 8 OR 9 OR 10 OR 11 |
| 13 | Prognos* [Title Abstract Keyword] |
| 14 | Survival analysis [Title Abstract Keyword] |
| 15 | Treatment outcome [Title Abstract Keyword] |
| 16 | Fatal outcome [Title Abstract Keyword] |
| 17 | Mortality [Title Abstract Keyword] |
| 18 | 13 OR 14 OR 15 OR 16 OR 17 |
| 19 | 4 AND 12 AND 18 |
