# Supplementary material for: Prognostic significance of Lymphocyte-activation gene 3 (LAG3) in patients with solid tumors: a systematic review, meta-analysis and pan-cancer analysis
Source: Cancer Cell Int. 2023 Dec 2;23:306. doi: 10.1186/s12935-023-03157-5 (PMC10693146; doi:10.1186/s12935-023-03157-5)
Supplement: Supplementary file 8 — Additional file 8: Table S2. Detailed quality assessment of case-control studies. [file 12935_2023_3157_MOESM8_ESM.docx]

**Table S2.** Detailed quality assessment of case-control studies.

| **Included Studies** | **Items of NOS** | | | | | | | | |
| --- | --- | --- | --- | --- | --- | --- | --- | --- | --- |
|  | **Selection** | | | | **Comparability** | **Exposure** | | | **Total** |
|  | Adequacy of the definition of the case | Representativeness of cases | Selection of the control | Definition of the control | Comparability of between cases and controls on basis of the design or analysis | Ascertainment of exposure | Whether exposures to cases and controls were determined using the same method | Nonresponse rate |  |
| Asano *et al.* 2022 | **★** | **★** |  | **★** | **★★** | **★** | **★** | **★** | 8 |
| Babar *et al.* 2019 | **★** | **★** | **★** | **★** | **★** | **★** | **★** | **★** | 8 |
| Baggudar *et al.* 2022 |  | **★** | **★** | **★** | **★★** | **★** | **★** | **★** | 8 |
| Bottai *et al.* 2016 |  | **★** | **★** | **★** | **★** | **★** | **★** | **★** | 7 |
| Chen *et al.* 2021 | **★** | **★** | **★** | **★** | **★** | **★** | **★** | **★** | 8 |
| Deng *et al.* 2016 | **★** | **★** | **★** | **★** | **★** | **★** | **★** | **★** | 8 |
| Duan *et al.* 2018 | **★** | **★** | **★** | **★** | **★★** | **★** | **★** | **★** | 9 |
| Fucikova *et al.* 2019 |  | **★** | **★** | **★** | **★** | **★** | **★** | **★** | 7 |
| Gebauer *et al.* 2020 |  | **★** | **★** | **★** | **★** | **★** | **★** | **★** | 7 |
| Giraldo *et al.* 2015 |  | **★** | **★★** | **★** | **★** | **★** | **★** | **★** | 8 |
| Guo *et al.* 2020 | **★** | **★** | **★** | **★** |  | **★** | **★** | **★** | 7 |
| He *et al.* 2017 | **★** | **★** | **★** | **★** | **★★** | **★** | **★** | **★** | 9 |
| Jin *et al.* 2023 |  | **★** | **★** | **★** | **★★** | **★** | **★** | **★** | 8 |
| Kim *et al.* 2018 | **★** | **★★** | **★** | **★** | **★** | **★** | **★** | **★** | 9 |
| Kim *et al.* 2020 |  | **★** | **★★** | **★** |  | **★** | **★** | **★** | 7 |
| Lee *et al.* 2018 | **★** | **★** | **★** | **★** | **★★** | **★** | **★** | **★** | 9 |
| Lee *et al.* 2019 | **★** | **★** | **★★** | **★** | **★** | **★** | **★** | **★** | 9 |
| Ligon *et al.* 2021 | **★** | **★** | **★** | **★** | **★** | **★** | **★** | **★** | 8 |
| Luo, C *et al.* 2021 | **★** | **★** | **★** | **★** | **★** | **★** | **★** | **★** | 8 |
| Luo, F *et al.* 2021 |  | **★** | **★** | **★** | **★** | **★** | **★** | **★** | 7 |
| Luo *et al.* 2022 | **★** | **★** | **★** | **★** | **★** | **★** | **★** | **★** | 8 |
| Lv *et al.* 2021 |  | **★** | **★** | **★** | **★** | **★** | **★** | **★** | 7 |
| Minichsdorfer *et al.* 2019 | **★** | **★** |  | **★** | **★** | **★** | **★** | **★** | 7 |
| Park *et al.* 2021 |  | **★** | **★** | **★** | **★★** | **★** | **★** | **★** | 8 |
| Peng *et al.* 2021 | **★** | **★** | **★** | **★** | **★** | **★** | **★** | **★** | 8 |
| Rhyner Agocs *et al.* 2021 | **★** | **★** | **★** | **★** | **★** | **★** | **★** | **★** | 8 |
| Rühle *et al.* 2022 |  | **★★** | **★** | **★** | **★** | **★** | **★** | **★** | 8 |
| Sarradin *et al.* 2021 | **★** |  | **★** | **★** | **★** | **★** | **★** | **★** | 7 |
| Seifert *et al.* 2021 |  | **★** | **★★** | **★** | **★★** | **★** | **★** | **★** | 9 |
| Shi *et al.* 2021 | **★** | **★** | **★** | **★** |  | **★** | **★** | **★** | 7 |
| Stovgaard *et al.* 2021 | **★** | **★** | **★** | **★** | **★** | **★** | **★** | **★** | 8 |
| Stovgaard *et al.* 2022 |  | **★** | **★** | **★** | **★★** | **★** | **★** | **★** | 8 |
| Tahtacı *et al.* 2023 | **★** | **★★** | **★** | **★** | **★** | **★** | **★** | **★** | 9 |
| Wang, H *et al.* 2019 |  | **★** | **★** | **★** | **★** | **★** | **★** | **★** | 7 |
| Wang, W *et al.* 2019 | **★** | **★** | **★** | **★** | **★** | **★** | **★** | **★** | 8 |
| Wang *et al.* 2018 | **★** | **★** | **★★** | **★** | **★** | **★** | **★** | **★** | 9 |
| Wang *et al.* 2021 | **★** | **★★** | **★** | **★** | **★** | **★** | **★** | **★** | 9 |
| Yao *et al.* 2023 | **★** | **★** | **★** | **★** | **★** | **★** | **★** | **★** | 8 |
| Zaitsu *et al.* 2023 | **★** | **★** | **★★** | **★** | **★** | **★** | **★** | **★** | 9 |
| Zeng *et al.* 2020 | **★** | **★** | **★** | **★** | **★** | **★** | **★** | **★** | 8 |
| Zhang *et al.* 2018 |  | **★★** | **★** | **★** | **★** | **★** | **★** | **★** | 8 |
| Zhang *et al.* 2022 | **★** | **★** | **★★** | **★** |  | **★** | **★** | **★** | 8 |
| Zou *et al.* 2023 |  | **★** | **★** | **★** | **★** | **★** | **★** | **★** | 7 |

A study can be awarded a maximum of one star for each numbered item within the Selection and Exposure categories. A maximum of two stars can be given for Comparability. Study rates ≥6 is eligible for further analysis. NOS, Newcastle-Ottawa Scale.
